# Electromagnetic Enantiomer: Chiral Nanophotonic Cavities for Inducing Chemical Asymmetry

**DOI:** 10.1021/acsnano.4c05861

**Published:** 2024-08-06

**Authors:** Rahul Kumar, Ben Trodden, Anastasia Klimash, Manon Bousquet, Shailendra K. Chaubey, Nicola J. Fairbairn, Ben A. Russell, Klaas Wynne, Affar S. Karimullah, Nikolaj Gadegaard, Peter J. Skabara, Gordon J. Hedley, Shun Hashiyada, Artur Movsesyan, Alexander O. Govorov, Malcolm Kadodwala

**Affiliations:** † School of Chemistry, Joseph Black Building, University of Glasgow, Glasgow G12 8QQ, U.K.; ‡ James Watt School of Engineering, Rankine Building, University of Glasgow, Glasgow G12 8LT, U.K.; § Innovative Photon Manipulation Research Team, RIKEN Center for Advanced Photonics, 2-1 Hirosawa, Wako, Saitama 351-0198, Japan; ∥ Department of Electrical, Electronic, and Communication Engineering, Chuo University, 1-13-27 Kasuga, Bunkyo-Ku, Tokyo 112-8551, Japan; ⊥ Department of Physics and Astronomy and Nano scale and Quantum Phenomena Institute, Ohio University, Athens, Ohio 45701, United States; # Institute of Fundamental and Frontier Sciences, University of Electronic Science and Technology of China, Chengdu 610056, China

**Keywords:** superchirality, strong coupling, chiral induction, nanophotonics, asymmetric chemistry, polaritonic chemistry

## Abstract

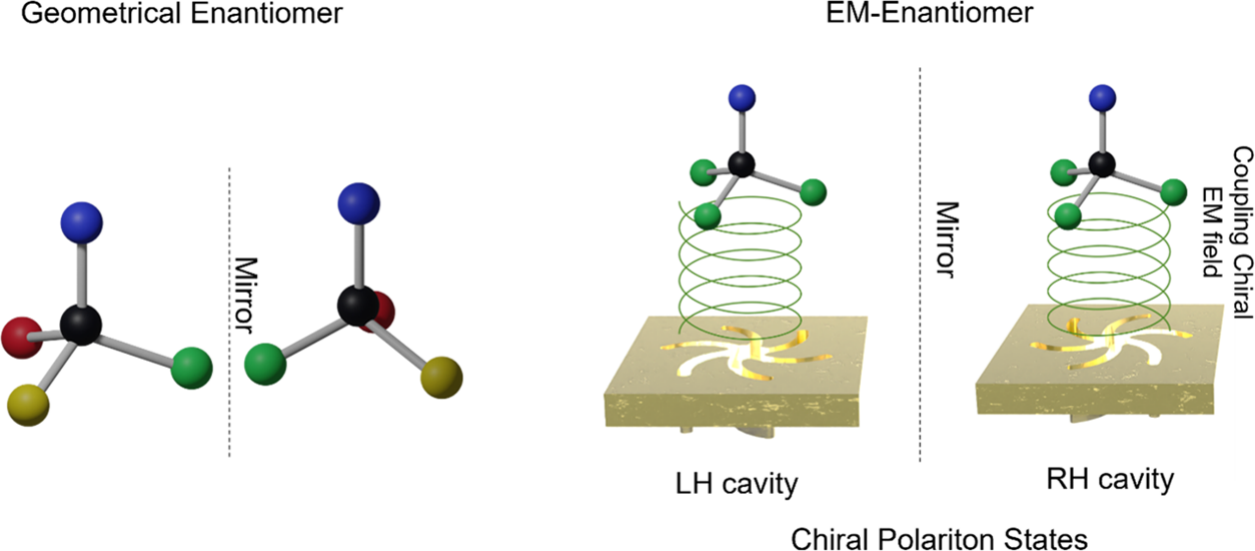

Chiral molecules, a cornerstone of chemical sciences with applications ranging from pharmaceuticals to molecular electronics, come in mirror-image pairs called enantiomers. However, their synthesis often requires complex control of their molecular geometry. We propose a strategy called “electromagnetic enantiomers” for inducing chirality in molecules located within engineered nanocavities using light, eliminating the need for intricate molecular design. This approach works by exploiting the strong coupling between a nonchiral molecule and a chiral mode within a nanocavity. We provide evidence for this strong coupling through angular emission patterns verified by numerical simulations and with complementary evidence provided by luminescence lifetime measurements. In simpler terms, our hypothesis suggests that chiral properties can be conveyed on to a molecule with a suitable chromophore by placing it within a specially designed chiral nanocavity that is significantly larger (hundreds of nanometers) than the molecule itself. To demonstrate this concept, we showcase an application in display technology, achieving efficient emission of circularly polarized light from a nonchiral molecule. The electromagnetic enantiomer concept offers a simpler approach to chiral control, potentially opening doors for asymmetric synthesis.

## Introduction

**Figure 1:**
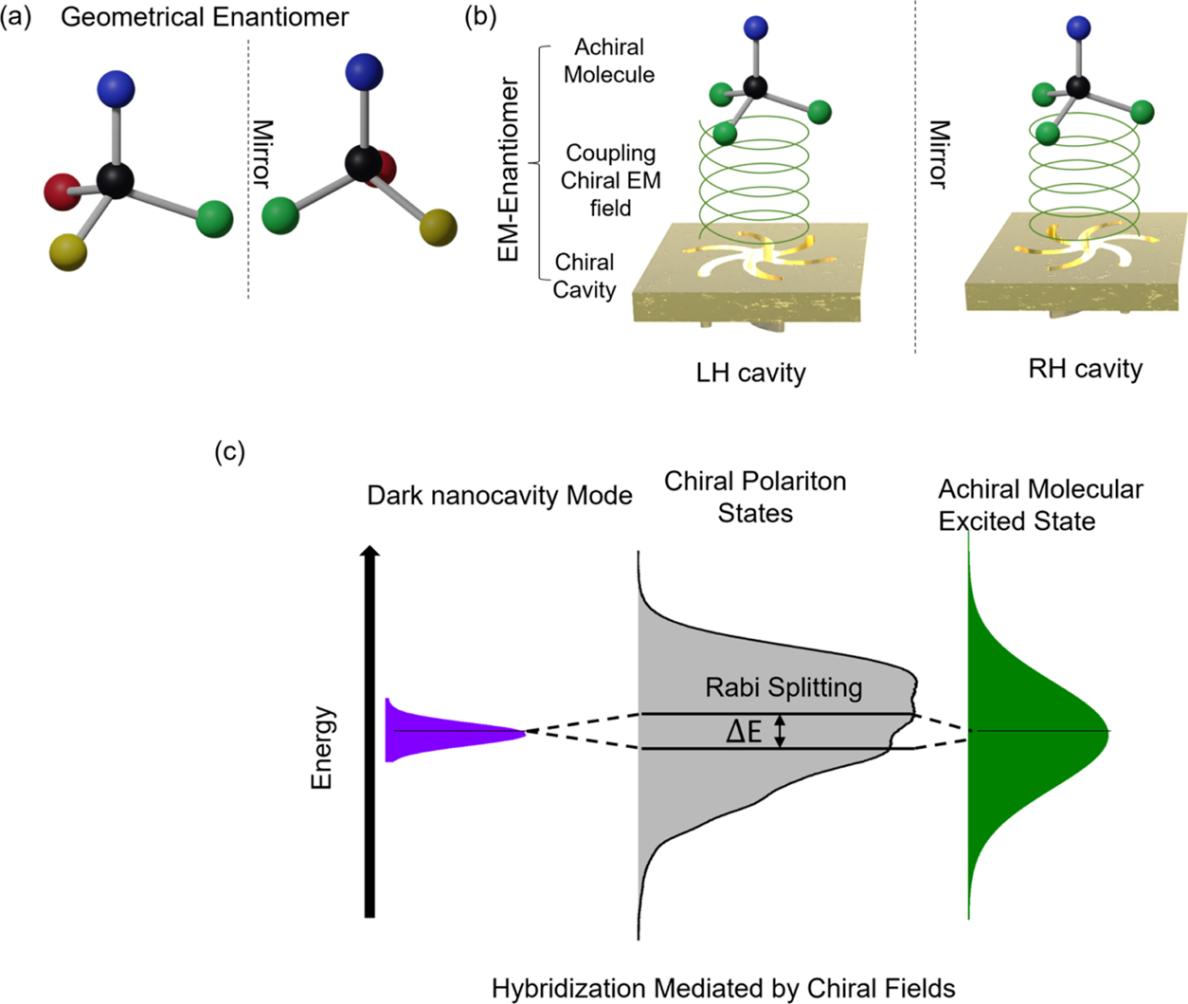
(a) Pair of enantiomers with mirror-image geometries. (b) Interaction of chiral near-fields with achiral molecules results in EM enantiomers through the symmetry-dependent creation of polariton states. (c) Schematic representation of strong coupling between the dark mode of an asymmetric photonic nanocavity and excited state of an achiral molecule placed within its vicinity, leading to the production of hybrid light–matter chiral polariton states.

Chirality, the property of asymmetry, is perceived as a geometric concept with chiral materials existing in otherwise identical mirror forms known as enantiomers. Thus, creating chiral molecules, which have applications spanning pharmaceuticals to next-generation photonic technologies, requires precise control of the three-dimensional (3D) structure, a challenging chemical problem. However, chirality is not just a physical property; it is also possessed by electromagnetic (EM) fields. Circularly polarized (CP) light is chiral and has long been used as a “chiral influence” to induce asymmetry in chemical reactions.^1−3^ Unfortunately, under normal conditions, CP light illumination cannot induce chirality in geometrically nonchiral (achiral) molecules, thus removing a potentially straightforward route to achieve chiral functionalities. In this work, we demonstrate a simple EM chiral induction mechanism that is based on strong coupling between the chiral dark mode of an asymmetric photonic nanocavity and the excited state of an achiral molecule placed within its vicinity, leading to the production of hybrid light–matter chiral polariton states. We refer to molecules chirally perturbed in this way as EM enantiomers, and this concept is illustrated in Figure 1. The level of coupling between the chiral near-fields and the molecule is dependent on the symmetries of both CP light and the chiral nanocavity, with symmetry match combinations leading to higher-intensity fields and hence strong coupling. We exploit this symmetry-dependent strong coupling to create a polymer film that has inherent photoluminescence with a high level of circular polarization (∼30%). This level of performance typically requires chiral molecular materials that have been complexly designed to display chirality on a macromolecular length scale.^4−6^ This illustrates how the EM-enantiomer concept could be used to simplify the design and construction and reduce the cost of organic light-emitting diode (OLED) sources of CP light, which are required for energy-efficient displays.

There are two extremes in the level of light–matter interactions: weak coupling, where the molecular excitation is unperturbed; and strong coupling, where hybrid electronic and photonic (polaritonic) states are created. Typically, those conditions can appear, when λ_mol_ ∼ λ_plasmon_ for a strong coupling, and when λ_mol_ and λ_plasmon_ are far from each other for the weak-coupling case. The formation of hybrid light–matter polaritonic states leads to the splitting of spectral bands, known as Rabi splitting.^7^ Strong and weak levels of coupling between electronically excited states and plasmonic modes have been reported in a range of nonchiral systems (i.e., the EM coupling fields have no chiral asymmetries).^8−10^ Few studies of coupling of achiral molecular excitation to chiral modes report only weak coupling.^11−13^ An experimental fingerprint of the strong coupling regime is the splitting of luminescent peaks (>50 meV).^8,10,14−17^ For achiral systems, strong coupling of excited molecular states to optical modes of cavities and antennae has been used to manipulate the selectivity of chemical reactions.^18^ This strategy is sometimes referred to as polaritonic chemistry and relies on the hybridization of states modifying activation barriers and altering excited-state lifetimes,^14,19−24^ thus altering reaction pathways.

In this work, we leverage the properties of the so-called dark chiral eigenmode, which are weakly dipole active, to facilitate strong coupling with chiral photonics modes of a chiral nanocavity. The wave functions (φ_EM–Enant_^±^) of electromagnetic enantiomers created by this hybridization of light–matter states are given by
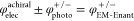
1where φ_elec_^achiral^ is the wave function of the achiral emitter state and φ_photo_^±^ are the wave functions of eigenmodes of the left (−)/right (−)-handed chiral nanocavities. The wave functions of the electromagnetic enantiomers are related through the parity operator (*P̂*)
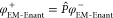
2with the two electromagnetic enantiomers being degenerate.

This work introduces the concept that strong chiral light–matter interactions can be used to imbue chiral properties onto a structural achiral molecule and hence can be a tool in chiral (asymmetric) chemistry, a previously unconceived paradigm of polaritonic chemistry.

## Results and Discussion

**Figure 2:**
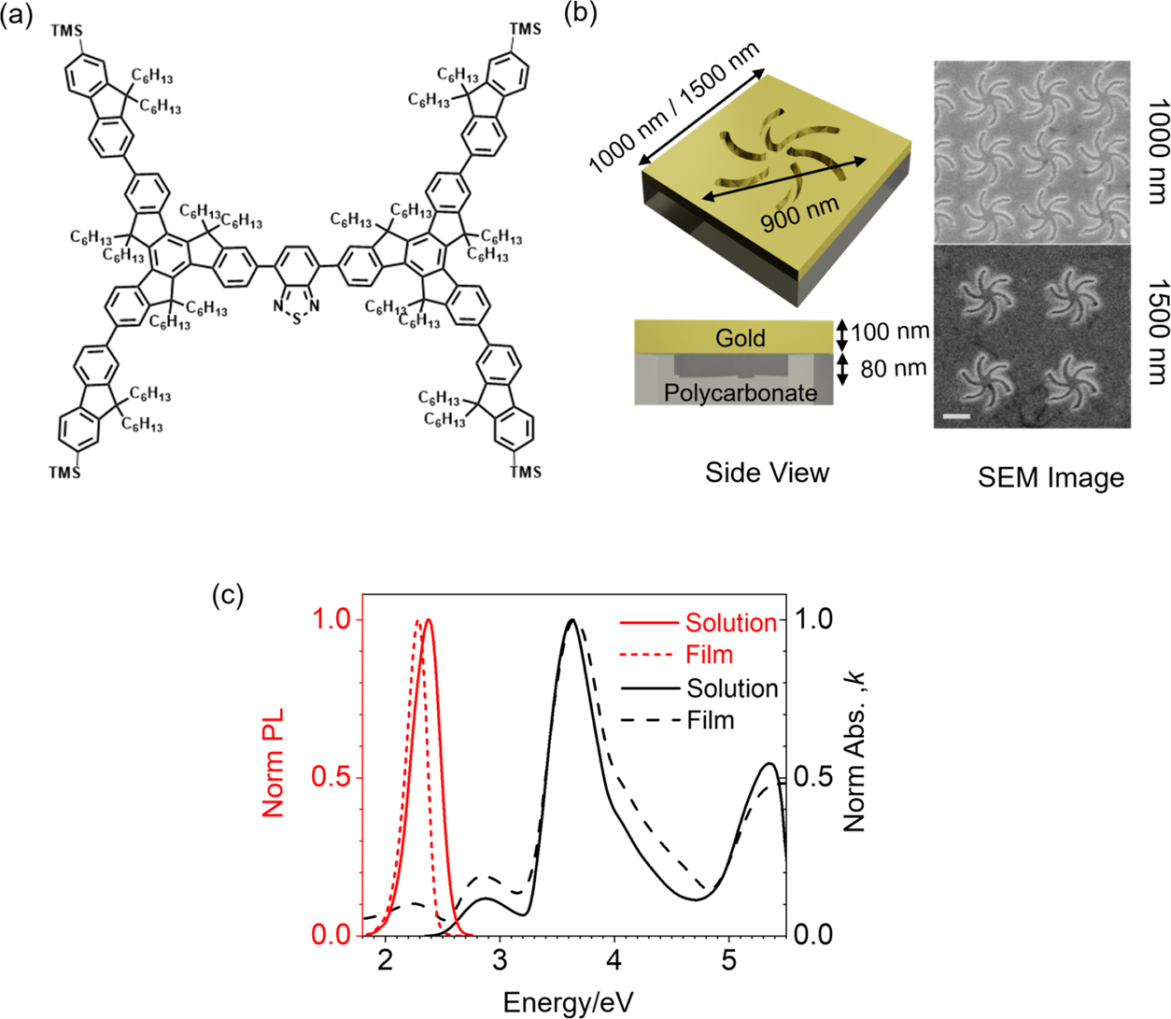
(a) Chemical structure of MHeB14. (b) Schematic showing orthographic (left top), side view (left bottom), and SEM images (right) of the RH nanocavity. The white scale bar at the bottom represents 500 nm on the micrograph. (c) Self-normalized absorbance (solid black) and luminescence (solid red) of MHeB14 in toluene, imaginary part of refractive index (dashed black), and luminescence (dashed red) of 50% (w/w) film.

We synthesized and used an emitter molecule 4,7-bis(7,12-bis(9,9-dioctyl-7-(trimethylsilyl)-9H-fluoren-2-yl)-5,5,10,10,15,15-hexahexyl-10,15-dihydro-5H-diindeno[1,2-a:1′,2′-*c*]fluoren-2-yl)benzo[*c*][1,2,5]thiadiazole (MHeB14), Figure 2a, which is a suitable material for use in OLEDs. Poly(methyl methacrylate) (PMMA) layers (∼200 nm thickness), which were either undoped or doped with 50% w/w of MHeB14, were spin-coated onto two types of nanostructured substrates. The substrates, which have been described in detail elsewhere,^25^ consist of ∼100-nm-thick Au films, deposited onto polycarbonate templates that contained periodic square arrays of either left- or right-handed shuriken indentations. We have used two types of templated structures; they both contain identical shuriken indentations, but with periodicities of 1000 and 1500 nm, respectively; see Figure 2b. Subsequently, the two spin-coated periodic cavities will be referred to as 1000 and 1500 nm metafilms. As will be shown later, although the shuriken structures are identical, these substrates have dissimilar EM environments. Effectively, they allow the EM environment to be modified without altering the structure of the nanocavity, mitigating against possible influences of changing the nanocavity on the polymer film structure.

### Optical Properties of MHeB114-Doped Polymer Films and Chiral Shuriken Nanocavities

**Figure 3:**
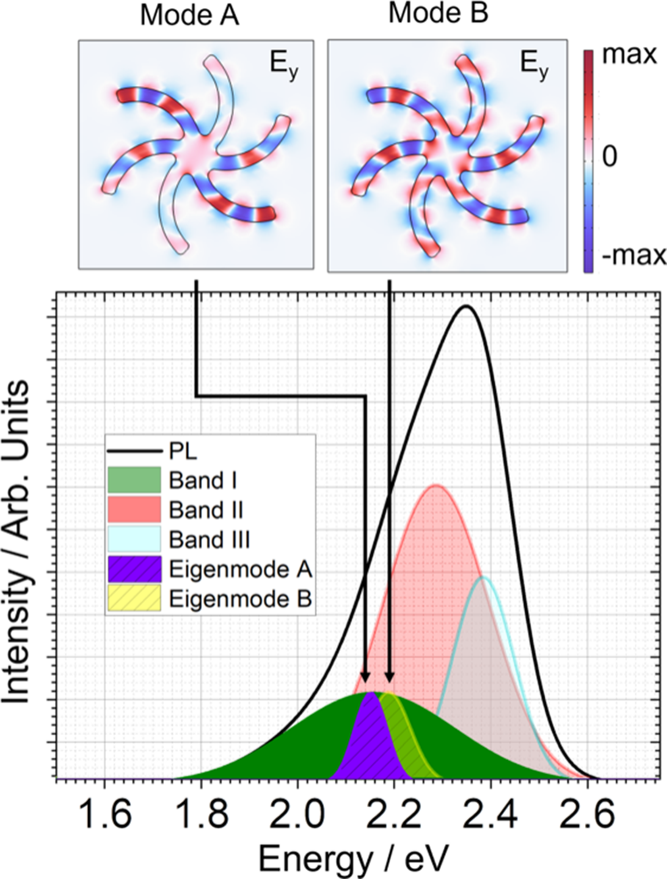
Three luminescence bands transitions, labeled as bands I, II, and III, are derived from Gaussian fitting of the photoluminescence (PL) spectrum of MHeB14 on a flat gold substrate. Two dark eigenmodes of nanocavities are show as violet and yellow solid line filled bands. The black arrows indicate the field profiles (top) illustrating the nondipolar *y*-component of the electric field associated with these modes.

For reference purposes, we have compared luminescence and absorption spectra for solutions of MHeB14 with the equivalent data from PMMA-doped films deposited on unstructured Au surfaces. This reference data, Figure 2c, demonstrates that the optical absorption and emission properties of MHeB14 immobilized in the PMMA are not significantly modified compared to that of the free molecule in solution, Figure 2c, which is indicative of an absence of molecular aggregate formation. The slight red shift of the luminescence compared to solution can be attributed to different dielectric environments and the reduced conformational flexibility of MBeH14 in the matrix. The luminescence spectra from doped films on unstructured Au films can be represented by three Gaussian components labeled I, II, and III, centered at 2.155, 2.286, and 2.385 eV, with corresponding line widths of 0.345, 0.219, and 0.128 eV, as shown in Figure 3. Fitting parameter details are given in Table S1. These luminescence bands, like those observed in related molecules, can be attributed to the vibronic structure associated with the excitation of C–C bonds.^26,27^

The optical modes of the shuriken nanocavities in the 1000 and 1500 nm doped metafilms were determined through two-dimensional (2D) eigenmode analysis. The complete list of calculated eigenmodes for 1000 nm doped metafilm, their line widths, and nature can be found in Table S2. The eigenmode analyses for both 1000 and 1500 nm metafilms yielded identical results, suggesting that the modes were localized on the shuriken nanocavities.

Two dark eigenmodes, denoted as modes A and B, have been identified with energies of 2.156 and 2.191 eV and widths of 0.090 and 0.106 eV, which overlap the luminescence bands I and II, respectively. The electric-field plots for modes A and B are illustrated in Figure 3. Both modes demonstrate field distributions in the plane of the metafilms (Figure 3) with centers of inversion signifying nondipole active multipolar character. As these multipolar eigenmodes exhibit weak interaction with plane waves incident perpendicular to the surface, a typical experimental condition, they can be categorized as dark modes. The extent of interaction between an electromagnetic (EM) field and multipolar modes relies on the magnitude of the field gradients.^28^ Consequently, EM fields that vary spatially over the length of the nanocavity interact more strongly with the multipolar mode. The calculated line widths of these dark modes are notably narrower than those experimentally observed for the inherently more lossy dipolar bright modes.^25^

**Figure 4:**
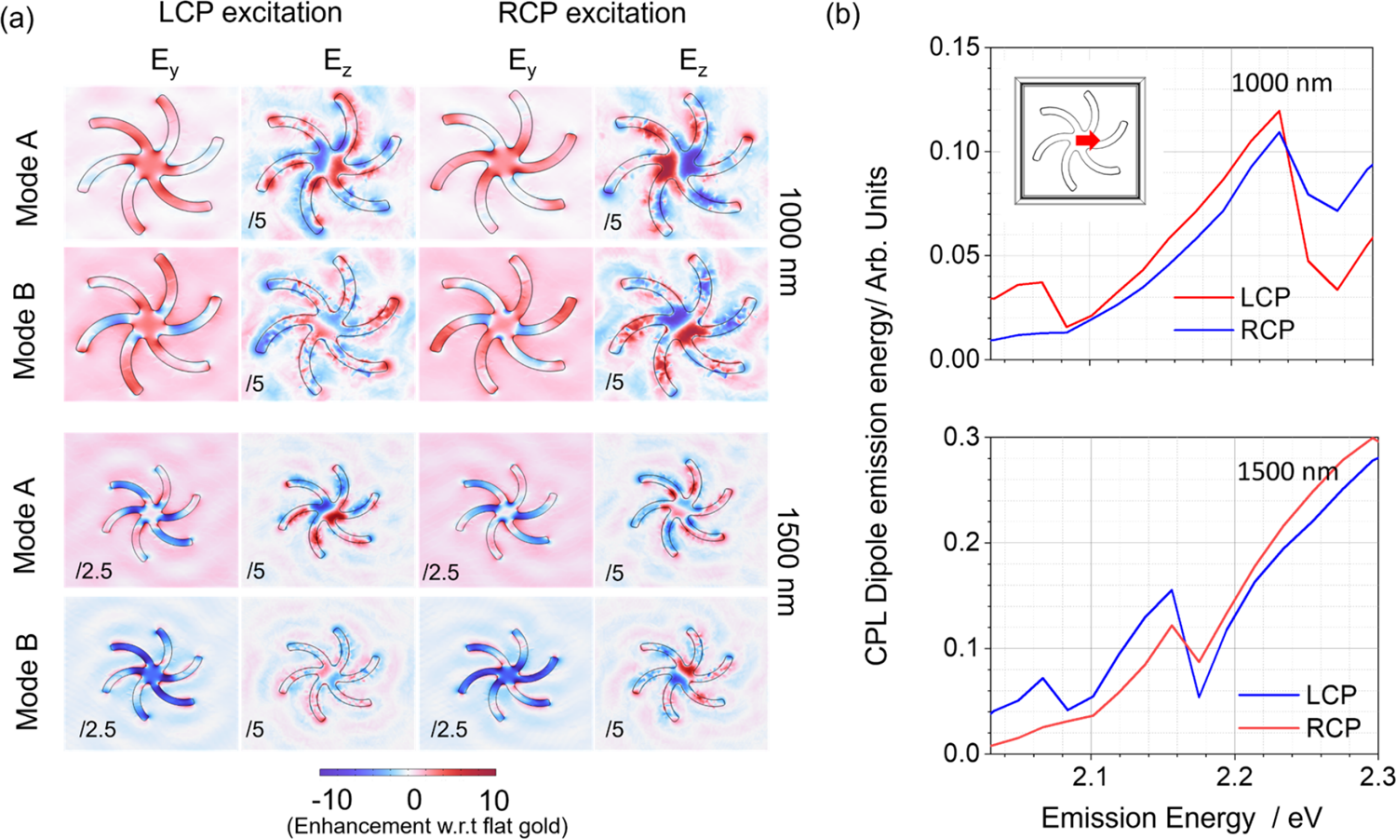
(a) In-plane (*E*_*y*_) and out-of-plane (*E_z_*) electric-field distributions for 1000 (top) and 1500 nm (bottom) metafilms at the center of the cavity at eigenmodes A and B. The incident E-fields are left and right circularly polarized, and the field values are normalized with respect to the corresponding values of flat gold. The numerical annotation at the bottom indicates the field scaling factor; for instance, “/2.5” implies that the maximum and minimum values are 2.5 times lower than the values indicated on the colorbar. (b) Averaged CPL emission from a single-point electrical dipole oriented at various angles and positioned at the center of the LH cavity is depicted for a metafilm at 1000 nm (top) and 1500 nm (bottom).

To provide quantitative insight into the nature of the EM environments, 3D numerical simulations were performed for enantiomorphic metafilms of both periodicities for normal incident plane wave illumination of left and right circularly polarized light (LCP and RCP). The simulations were performed on idealized models of shuriken nanocavities, which do not account for structural imperfections and defects, which can result in localized enhancement of both intensity and chiral asymmetry.^29,30^ These simulations were used to derive electric-field intensity maps at eigenmode energies. As shown in Figure 4a, the in-plane fields (shown here for *E*_*y*_) have nondipolar character but the field perpendicular to the surface (*E*_*z*_) is dipolar in nature (i.e., fields are odd under inversion). Moreover, the intensities of the fields are dependent on both the periodicity and helicity of light, with the 1000 nm metafilms having 2.5 times most intense in-plane fields. This is because the periodic square arrays in both metafilms can support in-plane diffractive lattice modes.^31,32^ These lattice modes amplify the electric near-field intensities in the vicinity of the nanocavities. For square arrays, lattice modes are characterized by parameters *n*_*x*_ and *n*_*y*_, representing the orders of diffraction in the *x* and *y* directions. The first 7 lattice modes are shown in Table S3. The relevant lattice modes in the vicinity of eigenmodes are the (2, 2) and (3, 3) modes for the 1000 and 1500 nm metafilms, respectively, both occurring at 2.384 eV (530 nm). Following diffractive behavior, the higher-order (3, 3) mode is expected to be less intense than the (2, 2) mode. Consequently, it is anticipated that the (2, 2) mode in the 1000 nm substrate would be associated with greater local field enhancements. However, symmetry-matched combinations of helicity and nanocavity (LCP/LH and RCP/RH) generate more intense fields than mismatched combinations (LCP/RH and RCP/LH) that can be seen from the E-field intensity profile given in Figure S8.

The helicity-dependent coupling and the presence of lattice modes can be further validated through the investigation of the circular polarization of luminescence (CPL) emission pattern of a single electric point dipole oriented at various angles and positioned at the center of the cavity. In Figure 4b, the averaged dipole emission is illustrated for metafilms at 1000 and 1500 nm. The figure reveals distinct dipole emission for different handedness, indicating an enhanced emission of one handedness over the other in certain energy bands. Moreover, these emission patterns vary as the periodicity of the metafilm shifts from 1000 to 1500 nm, thus indicating the presence of lattice modes. It is important to note that this simulation does not precisely replicate the experimental conditions where multiple coupled emitters emit simultaneously within the heterogeneous electromagnetic environment of the cavity. However, this simple simulation derived from ellipsometry macroscopic parameters provides compelling evidence of a CPL-dependent exciton–plasmon interaction.

The chirality of the shuriken nanocavity is instilled into its near-fields, which possess an inherent sense of asymmetry. The levels of chiral asymmetries of near-fields have been parametrized with the optical chirality factor (*C*)^33,34^ (see Figure S8). For both periodicities, there are local regions of space that have chiral asymmetries significantly larger (between 1 and 2 orders of magnitude larger) than that possessed by circularly polarized light, a property sometimes referred to as superchirality.^34−37^

To summarize, the optical cavities of the two metafilms have identical eigenmodes that spectrally overlap electronically excited vibronic modes of the MHeB14. The level of potential coupling between optical and electronic modes is modulated by two parameters, which influence the intensities of the electric near-field environments. The lower-order lattice mode provides a greater enhancement for the 1000 nm case. In addition, the intensities of the electric field are helicity-dependent, with the most intense fields generated by symmetry-matched combinations of helicity and cavity handedness. Consequently, the most favorable case for observing strong coupling is for symmetry-matched combinations of helicity and handedness for the 1000 nm metafilms.

### Helicity-Dependent Luminescence and Reflectance from Metafilms

**Figure 5:**
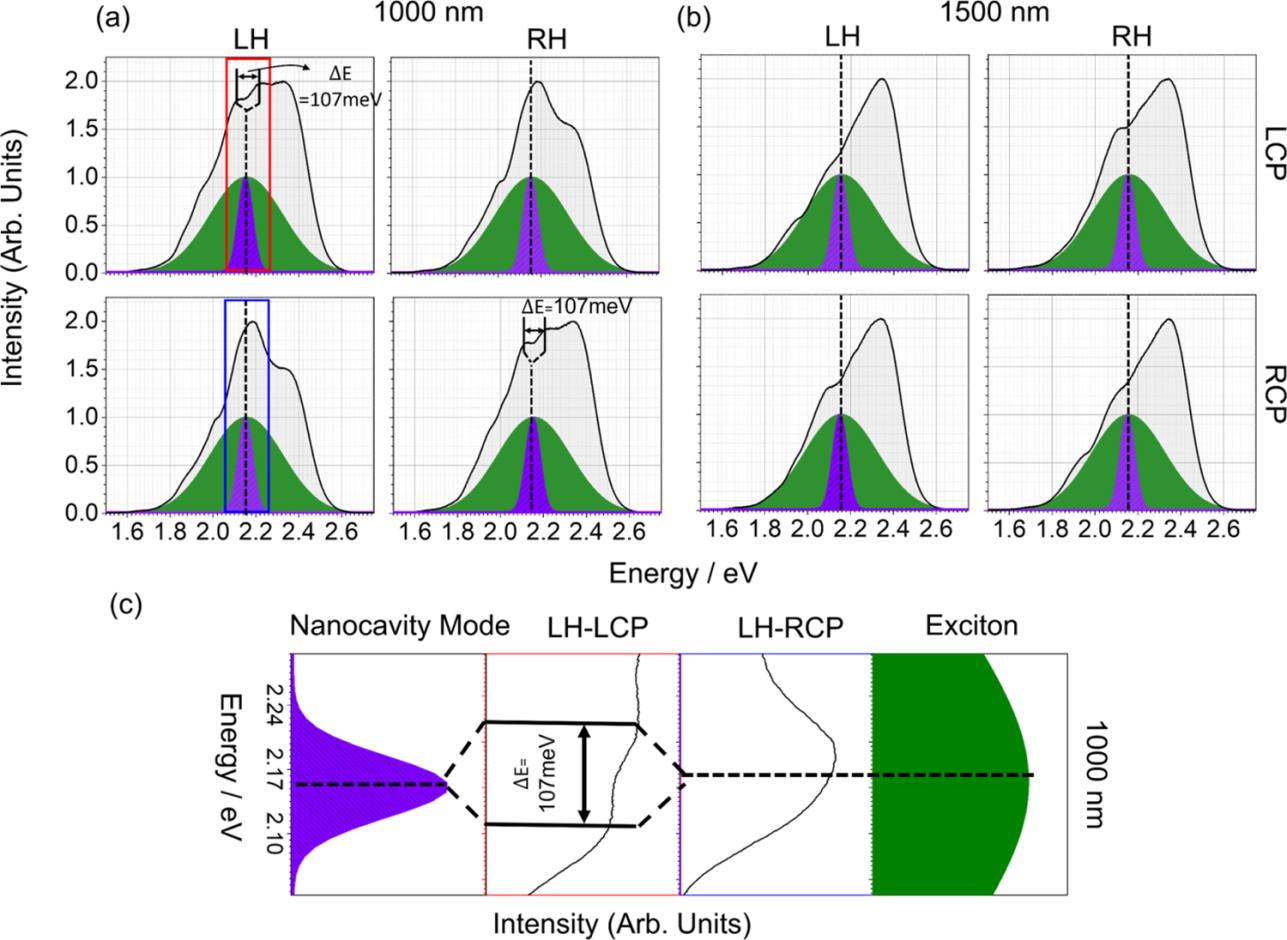
Left circularly polarized emission (top) and right circularly polarized emission (bottom) for (a) 1000 and (b) 1500 nm metafilms. The olive and blue colored solid dash filled spectra correspond to luminescence band I of molecules on flat gold and dark eigenmode A of nanocavity. The dashed black vertical lines are guidance for eyes, highlighting the correspondence between PL peak, maxima of dark eigenmode and luminescence band I. Δ*E* shows the rabi splitting of energy level for the matched combination. (c) Enlarged version of LH-LCP and LH-RCP spectra, marked as red and blue boxes in the original graph, showing PL peak splitting and enhancement for the matched and mismatched combinations, respectively.

Luminescence from both enantiomorphs of 1000 and 1500 nm doped metafilms for LCP and RCP emission is shown in Figure 5a,b, respectively. The line shapes of the luminescence spectra from the 1000 nm metafilm are strongly helicity-dependent. For both matched and mismatched combinations of helicity and cavity handedness, there is the structure that coincides with the spectrally overlapping molecular band I and eigenmode A. In the matched case, there are two discernible peaks that are located at either side of this point with the peak-to-peak separation of 107 meV as shown in zoomed Figure 5c. However, for the mismatched combinations, a single peak is observed at this position. The 1500 nm doped metafilms display much weaker helicity dependency, with a shoulder observed at the position of the overlapping eigenmode A and band I for both matched and mismatched cases, albeit slightly more pronounced for the former. For both periodicities of metafilms, there are also less intense structures <2.0 eV that are derived from emission that overlaps plasmonic modes of the nanostructure.

The relative reflectance spectra, shown in Figure S9 for both periodicities, display helicity dependence, with once again symmetry-equivalent combinations of CPL and cavity handedness giving identical spectra. The spectra are dominated by features associated with the plasmonic resonances <2.0 eV. However, there are weaker features that coincide with the overlap of eigenmode A and band I. For mismatched combinations, there is a slight dip in reflectance, and for matched combinations the opposite is observed with a slight peak in reflectance.

### Circular Dichroism in Luminescence and Reflectance (CPL and CD)

A comparison is made between dichroic spectra derived from the scattering and luminescence of circularly polarized light, which in accordance with the literature are plotted in terms of the asymmetry factor (*g*) and will be referred to as circular dichroism (CD) and CPL spectra, respectively. Spectra were derived from data for doped and undoped metafilms of both enantiomorphs and periodicities, as shown in Figure S10. It should be noted that the luminescence from the undoped films is derived from the radiative decay of holes in the 5d band of gold, which produces very weak emission (two orders of magnitude weaker than doped PMMA films) spanning the blue to IR regions of the spectrum.^38^

In the case of the undoped films, the CPL and CD spectra are quantitatively similar. This would be expected, since in the absence of potential coupling to an excited molecular state, both the CPL and CD responses will reflect the EM of the shuriken cavities.^39^

As expected from the individual spectra, the 1000 nm metafilms show a peak in both circularly polarized luminescence (CPL) and circular dichroism (CD) spectra, positioned at the energy where eigenmode A and luminescence band I overlap. However, there is a difference in the polarity of this peak in the CPL and CD spectra. This reversal of polarity can be explained by the association of luminescence peaks with discrete states; transitions leading to luminescent peaks exhibit equivalent features in absorption, resulting in absorption peaks causing dips in the reflectivity. This positive–negative relationship between luminescence and reflectance peaks accounts for the reversal of the dichroic peak’s polarity.

The implication of this is that the reflectance spectra exhibit equivalent split-single resonances for matched–mismatched combinations, similar to those observed in luminescence. However, these features in reflectance are small compared with the more intense responses associated with plasmonic modes. In contrast, the CPL and CD spectra from the 1500 nm metafilms do not exhibit a similar common resonance in the regions of eigenmode A and band I.

To recap, the luminescence, reflectance, and dichroic data are consistent with strong coupling between the electronic excited state and a dark mode of the shuriken cavity for matched combinations at 1000 nm for the MHeB14 molecules embedded within the polymer matrix. Specifically, the observed peak splitting in both luminescence and reflectance, confirmed by the shared dichroic resonances in CD and CPL, signifies strong coupling. While peak splitting in extinction spectra may arise from various mechanisms aside from strong coupling, such as Fano interference,^40^ equivalent splitting in luminescence provides a distinct signature of strong coupling.^16^ This distinction arises because photoluminescence (PL) operates as an incoherent process, unlike light scattering and thus does not exhibit Fano interference. In contrast, the solitary luminescence/reflectance peak observed from the 1000 nm metafilm for mismatched combinations can be attributed to the Purcell effect,^41^ a phenomenon occurring in the weak-coupling regime, affecting a fraction of the molecules within the cavity. This effect is facilitated by the presence of eigenmode A, leading to an increase in the local density of states (LDOS) within specific regions of the shuriken cavity. The luminescence and scattering data obtained from the 1500 nm metafilms, whether in matched or mismatched combinations, can also be interpreted within the Purcell framework. The shoulder observed in the luminescence spectrum originates from the enhanced LDOS within cavity regions influenced by eigenmode A. The ability of dark modes of nanostructures/nanocavities to strongly couple to the dipole emitter has been established in achiral systems.^42,43^

### Emission Angular Distributions

Established methods for validating strong coupling rely on fitting experimental spectra to models that replicate energy Rabi splitting.^16,17^ However, this approach assumes a uniform environment for all emitters, which becomes problematic in complex systems with molecules. Here, emitters can experience a range of coupling strengths from weak (Purcell effect) to strong. Consequently, a single measured spectrum becomes a mixture of contributions from these various environments. Fitting such a “multicomponent” spectrum to a single strong coupling model can be misleading, potentially suggesting strong coupling even when many emitters are weakly coupled. This is where measuring and modeling emission angular distribution patterns offer a significant advantage. These patterns reveal the spatial distribution of the emitted light intensity. For systems exhibiting strong coupling, the angular distribution deviates significantly from the pattern expected for a weakly coupled system, even if the coupling strength varies across different emitters. By comparing the measured angular patterns with theoretical models that account for strong coupling, one can gain a more robust validation of the phenomenon in heterogeneous systems. In essence, analyzing emission angular patterns provides independent information beyond just energy splitting. It offers a clearer picture of light–matter interactions within complex environments, making it a more reliable tool for confirming strong coupling in systems in which the coupling strength varies.

Consequently, to provide further evidence on the nature of the coupling between MHeB14 and the cavity, we collected helicity-dependent angular distributions of emitted light within an energy window (with of 0.074 eV, centered at 2.138 eV) that covers the overlap of band I and eigenmode A. The angular distributions were obtained using spectroscopic back focal plane imaging.^44^ The angular distribution of emission reflects the level of coupling between the emitter and the can cavity. In the absence of an optical cavity, the randomly oriented molecule will emit photons isotropically over a 2π azimuthal angle with uniform intensity and no structure. Consequently, the observation of structured angular emission distributions indicates a level of coupling between the emitter and the cavity.

**Figure 6:**
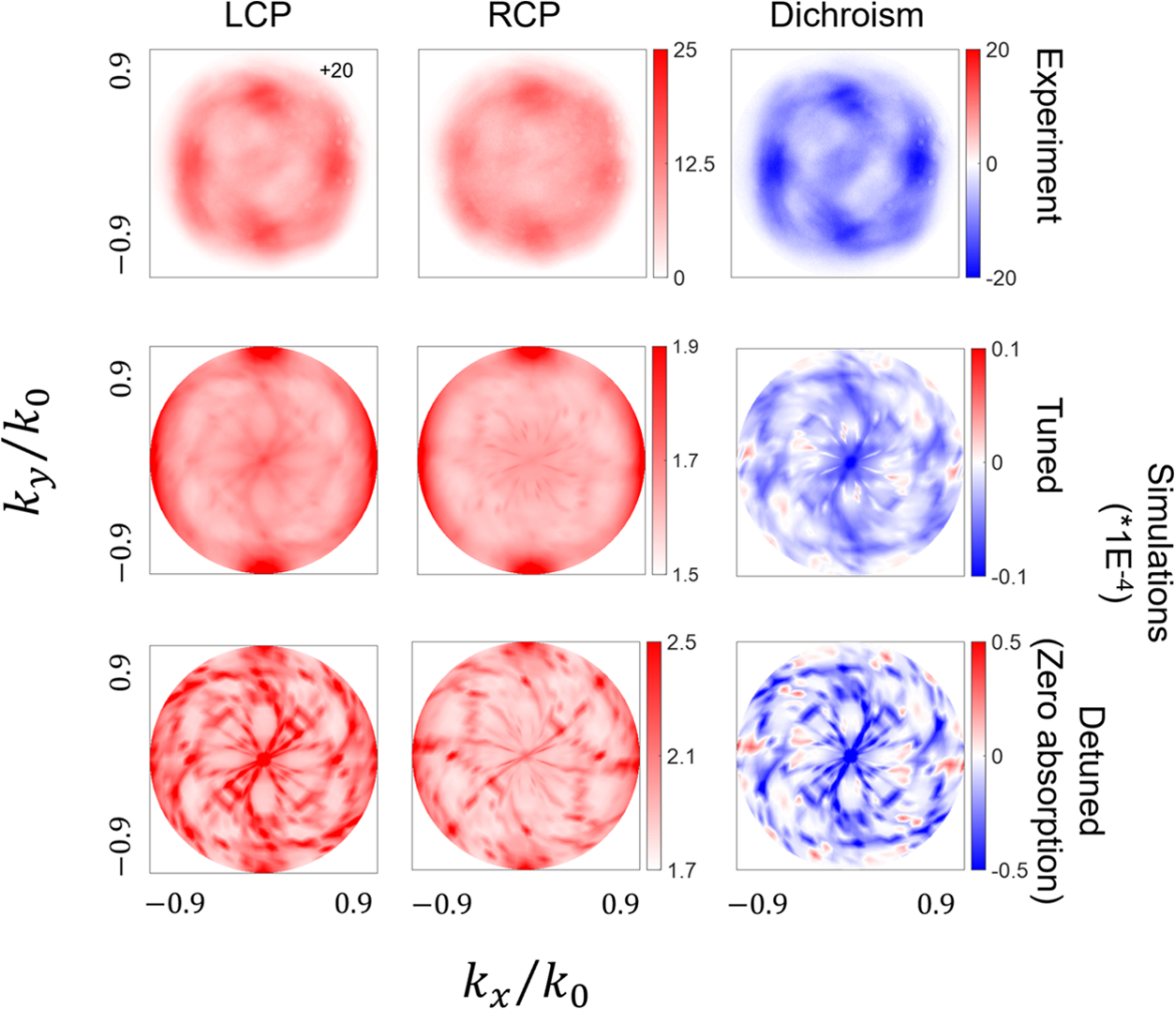
Experimental and simulated angle-resolved LCP and RCP emission along with dichroism (LCP–RCP) of the MHeB14-doped LH 1000 nm metafilm. In the experiment, the colors represent the relative intensity count with respect to the flat background. In the simulation, the colors represent the volume-integrated E-field magnitude in the 200 nm film.

**Figure 7:**
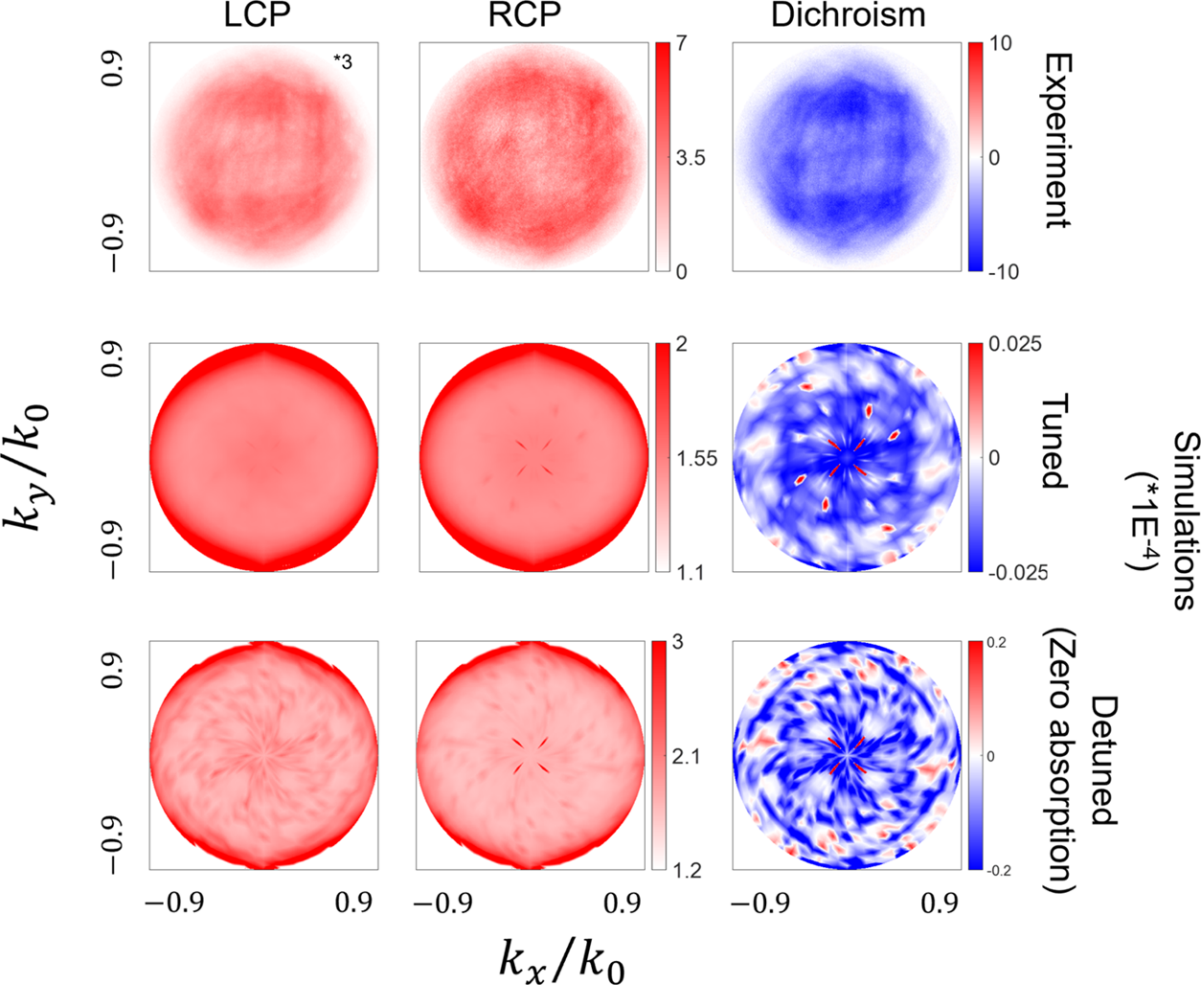
Experimental and simulated angle-resolved LCP and RCP emission along with dichroism (LCP–RCP) of the MHeB14-doped LH 1500 nm metafilm. In the experiment, the colors represent the relative intensity count w.r.t. the flat background. In the simulation, the colors represent the volume-integrated E-field magnitude in the 200 nm film.

Figures 6 and 7 illustrate the angular distribution patterns for both enantiomorphs of 1000 and 1500 nm LH metafilms for LCP and RCP emission, and the dichroic difference (LCP–RCP). The angular distribution patterns of RH metafilms are shown in Figure S11.

For the 1000 nm metafilms, both matched and mismatched combinations exhibit helicity-dependent structured angular distributions, with mirror-image distributions obtained for combinations of helicity and cavity handedness that are symmetry equivalents. The left circularly polarized (LCP) and right circularly polarized (RCP) angular distributions of matched and mismatched combinations show a common structural component: four lobes of greater intensity at high emission angles (38°), occurring at azimuthal angles of 0, 90, 180, and 270°. These lobes become more apparent in the dichroic angular distribution pattern. We propose that these 4-fold symmetric lobe structures are associated with the square lattice of the metafilm array. This interpretation aligns with the previous argument, suggesting that the lattice modes of the array perturb the near-field environment associated with eigenmodes of the shuriken.

The appearance of lobes at larger emission angles suggests that circularly polarized photons are coupling to the dipole character possessed by the dark eigenmode A of nanocavity in the *z*-direction. Additionally, the angular emission patterns for matched combinations, but not for mismatched ones, display an “S”-like structure connecting lobes at 0 and 180°. Consequently, the angular distribution patterns of matched combinations exhibit a greater degree of mirror symmetry breaking than those of mismatched combinations, indicating a higher level of coupling to the chiral shuriken cavity.

In contrast, the 1500 nm metafilms demonstrate more isotropic angular distributions with no noticeable helicity dependence. However, emission at higher angles remains favored. The absence of a strong angular structure, the relatively isotropic emission, and the lack of mirror symmetry breaking in the pattern are consistent with a weaker level of coupling between the emitter and the shuriken cavity.

Indeed, the angular emission patterns correlate with the behavior observed in the luminescence data. For the 1000 nm metafilms, stronger coupling is observed in the matched combinations compared with the mismatched combinations. Conversely, for the 1500 nm metafilms, the patterns are consistent with a weaker level of coupling than that observed for either helicity in the 1000 nm metafilms.

### In Silico Detuning

Although peak splitting is indicative of strong coupling, it is not typically solely relied upon in the literature for attributing strong coupling. Instead, a preferred strategy involves “detuning” the molecular and cavity resonances to definitively assign strong coupling. As the cavity and molecular resonances are detuned progressively, the level of hybridization decreases, leading to the observation of anticrossing.^22,45^ Implementing this strategy in complex cavities, such as the shuriken, necessitates synthesizing a range of molecules where the excited-state energy is systematically shifted without significantly altering the transition dipole strength or other material properties. While achievable for some classes of simple molecules, this task becomes more challenging, if not impossible, for complex molecular systems like MHeB14. Conversely, detuning the cavity mode is of limited effectiveness for complex cavities, since eigenmode energies cannot be systematically tuned without altering the overall electromagnetic environment. Thus, the application of this criterion to polariton formation is constrained by chemical synthesis limitations.

An alternative approach to address these chemistry-related limitations is conducting in silico experiments. Numerical simulations, based on classical electromagnetics, provide a means to replicate strong coupling.^45^ For instance, adjusting the separation between the emissive state of MHeB14 and the dark eigenmode is easily achievable through shifting the peak associated with the emissive state in the experimentally measured (ellipsometry) imaginary part of the dielectric response of the PMMA-doped film used in the simulation.

Figures 6 and 7 depict simulations of the helicity-dependent angular distribution patterns for both 1000 and 1500 nm metafilms. These simulations represent the “tuned” case, where the emissive state and dark eigenmode spectrally overlap. To isolate the influence of light absorption, we performed additional simulations (“detuned” case) where the resonance in the imaginary part of the dielectric response is removed, effectively eliminating coupling between the molecule and the cavity.

It is important to acknowledge that the simulations were conducted at a single energy (2.138 eV) and that the real part of the dielectric response replicates experimental observations. Consequently, both the “tuned” and “detuned” cases share identical refractive index properties, differing only in the magnitude of optical losses.

While this approach simplifies the model by neglecting the Kramers–Kronig relationship (which links the real and imaginary parts of the dielectric function), it serves a specific purpose. We aim to clearly demonstrate the dependence of angular emission patterns on optical absorption (imaginary part) specifically. In a weak-coupling regime, light scattering, governed by the real part of the dielectric function, would dominate the angular emission pattern. While simulations were performed at a single photon energy, experimental data were collected using a band-pass filter with a width of 20 nm, centered at 580 nm. This implies that any experimentally observed angular structure would be less well-resolved (i.e., less sharp) than in the “monochromatic” simulations.

Tuned simulations of 1000 nm metafilms qualitatively replicate the experimentally observed patterns, specifically the presence of four lobes and the mirror symmetry breaking “S” structure of the matched combination. Conversely, simulations for 1500 nm metafilms accurately capture the lack of significant helicity dependence in angular distribution as well as the helicity dependence of relative intensities in angular distributions.

Detuning has a significant impact on the angular distributions of 1000 nm metafilms, leading to the disappearance of the four well-defined lobes. Similar angular distribution patterns are observed for the matched and mismatched combinations, although there is weaker contrast in the former case. Significantly, qualitatively, the patterns for the matched combination display weaker mirror symmetry breaking than that in the tuned simulation. This is consistent with weaker coupling of the excited state to the chiral cavity mode. These outcomes differ from the effects of detuning on the simulations for the 1500 nm metafilms, where there is no substantial change in the overall angular distribution patterns. The primary distinction lies in the detuned data, where there is a more pronounced contrast between regions of minimum and maximum intensities, resulting in more resolved patterns.

We propose that the significant effects of simulated detuning on the angular distributions support the assumption that strong coupling occurs in the matched combination of the 1000 nm metafilms. However, the relatively small effects of detuning on the simulated patterns for 1500 nm metafile evidence weak coupling.

Consequently, the experimental and modeled angular distributions provide additional support for the inferred relative coupling strength derived from the luminescence and scattering spectra.

### Fluorescence Lifetime Imaging Microscopy Data

To further substantiate the presence of strong coupling between the excited state of the molecular emitter MHeB14 and a dark chiral cavity mode, we conducted fluorescence lifetime imaging microscopy (FLIM) measurements. These measurements provide detailed insights into the luminescence lifetimes of emitters under different conditions. The fluorescence lifetimes were derived from the total luminescence yield of MHeB14, encompassing all emissive wavelengths and without polarization selectivity. This approach ensures a comprehensive comparison between the luminescence lifetimes and intensities of MHeB14 in two different substrates with feature sizes of 1000 and 1500 nm and a reference flat Au surface.

**Figure 8:**
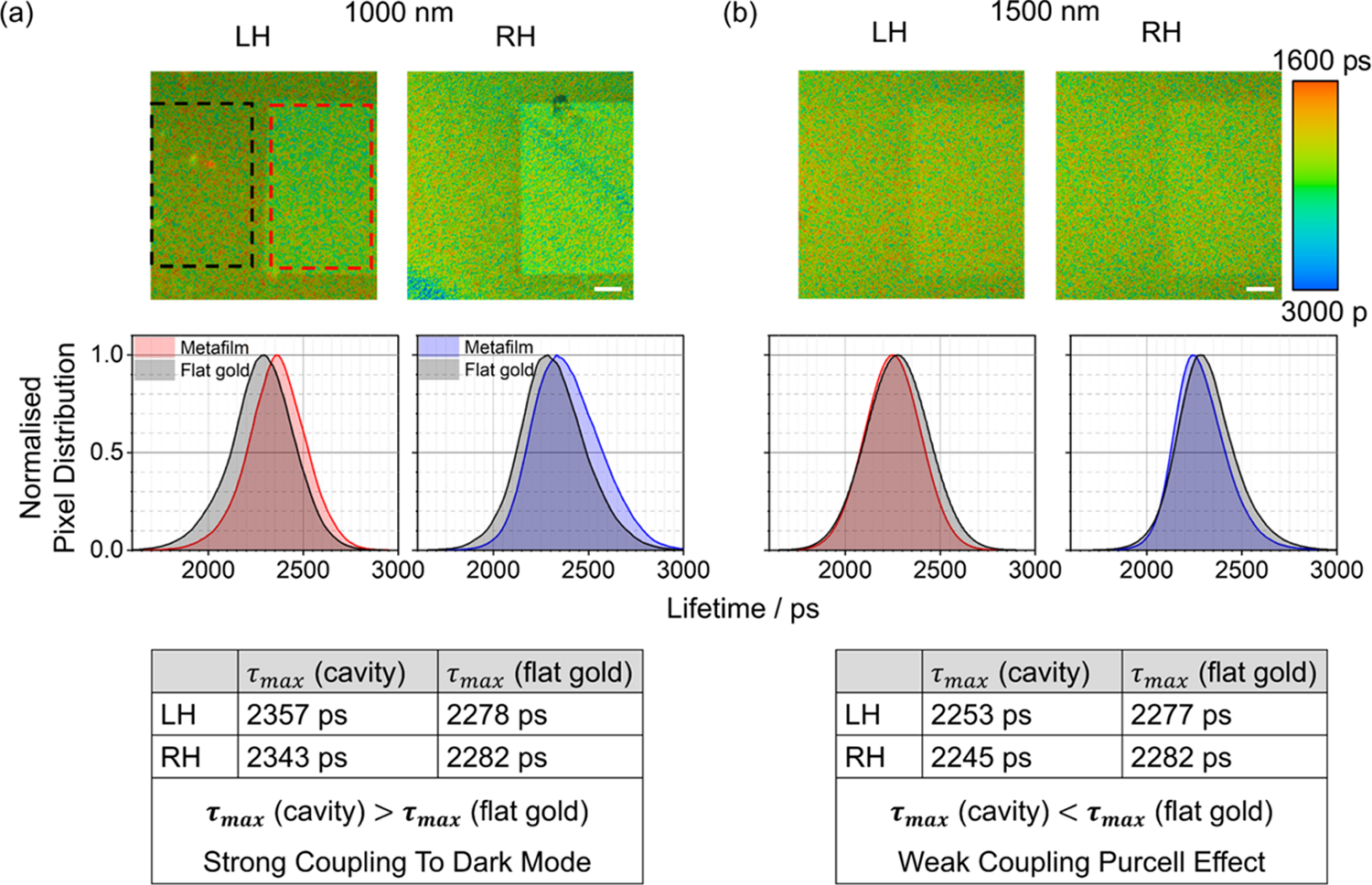
Fluorescence lifetime spatial map for MHeB14 molecules (top) and the distribution of lifetimes obtained from the highlighted area—black dashed box for molecules on flat gold and red dashed for structures—(bottom) for (a) 1000 and (b) 1500 nm metafilms. τ_max_ corresponds to the lifetime of the peak of normalized pixel distribution. The white scale bar at the bottom on lifetime images corresponds to 50 μm.

Figure 8 shows the spatial map lifetimes for the MHeB14 molecules in the 1000 and 1500 nm substrates for LH and RH structures and surrounding backgrounds. In the context of weak coupling, the Purcell effect typically manifests as an enhancement in both the rate and intensity of emission. This enhancement is quantified by the Purcell factor (*F*_P_), which can be expressed as
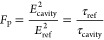
3where *E*_cavity_^2^ and *E*_ref_^2^ represent the electric-field intensity within the emission volume for the cavity and reference, respectively, and τ_cavity_ and τ_ref_ are the luminescence lifetimes for the cavity and reference configurations. Given the near-field enhancements present in the shuriken-shaped cavity (as in Figure S12), the *F*_P_ is expected to be greater than 1 (*F*_P_ > 1). This implies that, under the influence of the Purcell effect, both the intensity and emission rate of the emitter, MHeB14, should be increased, leading to a decrease in the fluorescence lifetime.

The lifetime distributions for the 1500 nm arrays shows a shift toward shorter times, consistent with the Purcell effect. However, a distinct behavior is observed for the 1000 nm substrate. Although the emission intensity from the arrays is enhanced, indicating a possible near-field effect, the lifetime of the MHeB14 emitter in these arrays is significantly longer than that of the molecules outside the cavity. This observation is inconsistent with the typical Purcell effect, which would predict shorter lifetimes in the presence of an enhanced emission. The extended lifetime in the 1000 nm cavities suggests a different interaction mechanism. This behavior is consistent with our proposal of strong coupling of the emissive molecular excited state and the chiral dark mode of the shuriken cavity. Intuitively, one would expect that hybridization of a bright radiative mode with a dark weakly radiative mode would result in a longer lifetime. Indeed such an increase in lifetime has been previously reported in plasmonic systems where dipole modes are strongly coupled to dark modes.^46^ Therefore, the observed increase in the lifetime of the emitters in the 1000 nm cavities relative to that of the free molecules provides further evidence for the strong coupling between an electronically excited molecular state and a dark chiral cavity state.

In summary, the formation of hybrid light–matter polariton states on 1000 nm substrates is attributed to two key observations: the dependence of the emission’s angular distribution on the molecular absorption properties, and the significant increase in the molecules’ luminescence lifetime inside the cavity. These factors together indicate strong coupling between the molecular states and a chiral dark optical mode of the shuriken cavity, confirming the creation of hybrid polariton states, which display the spectroscopic signatures of chirality.

## Conclusions

We have successfully demonstrated the manipulation of symmetry in the light–matter interaction, leading to hybrid light–matter states. This accomplishment was realized by strongly coupling a dark chiral mode of a nanocavity with an excited state of an achiral emitter molecule. Our investigation highlights the innovative utility of harnessing chiral dark modes for the generation of chiral polaritons. The inherent characteristics of dark modes, characterized by low intrinsic losses and high spatial field localization, play a pivotal role in facilitating strong coupling. The emitter molecule exhibits distinct luminescence behaviors under conditions of strong coupling, featuring peak splitting, and under weak coupling, enhanced emission attributable to the Purcell effect.

This study draws a valuable comparison to previous work demonstrating that chiral molecular layers can induce chirality in achiral nanofabricated Au plasmonic structures without requiring structural modification.^47,48^ Specifically, this process occurs through the interaction between plasmon polaritons and the surrounding chiral dielectric environment. The mechanism behind this phenomenon involves the real part of the dielectric function of the chiral molecular layer, which plays a crucial role in imparting chirality to the plasmon polariton. These findings illustrate how an inherently achiral system can acquire chiral properties via interactions with its chiral environment. Analogously, our study shows how an initially achiral emitter molecule can gain chiral characteristics through the formation of a hybrid light–matter polariton state. This transformation follows similar principles, where the interaction between the molecular excited state and the photon field leads to the emergence of chiral properties, even in the absence of intrinsic structural chirality in the molecule.

While electromagnetic enantiomer states are inherently degenerate (having the same energy), fascinating phenomena emerge when the achiral emitter molecule possesses prochirality. Prochiral molecules can be transformed into chiral ones, with either left- or right-handed configurations while retaining their light-absorbing group (chromophore). This conversion can be achieved, for example, by modifying a substituent group in a specific way. Strong coupling between the now-chiral emitter and the photonic eigenmode of the nanocavity will persist after the conversion, since the chromophore remains unaffected. However, the resulting hybrid states now hold the potential to form four distinct diastereomer states. These states can be described by the following wave functions



Among these four states, two pairs are actually enantiomers related through the parity operator (*P̂*). Here is the relationship between these enantiomeric pairs



The remaining two pairs are classified as symmetry-inequivalent diastereomers. Unlike enantiomers, these diastereomers are not related by the parity operator
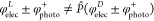


Crucially, these symmetry-inequivalent pairs are not degenerate, meaning the hybrid energy levels have differing energies, which has a significant implication. Depending on the specific handedness (left or right) of the nanocavity, one of the diastereomer states formed from the reaction with the prochiral species will be more stable and, therefore, preferentially formed. This selectivity can be controlled by using a nanocavity of opposite handedness, leading to the production of the chiral product with the opposite configuration.

We assert that designing a cavity with the appropriate optical modes for creating a chiral polariton is more feasible and versatile than employing asymmetric synthesis to generate chiral molecules. This method not only simplifies the experimental setup but also enhances the precision and control over the chiral interaction dynamics.

This approach offers an innovative parallel to the well-established method of using diastereomers for asymmetric synthesis.^49^ However, instead of traditional chemical methods, it utilizes light–matter interactions within engineered cavities. This concept, embodied by the “electromagnetic enantiomer”, presents a potentially simpler route for introducing asymmetry into chemical systems. By relying on a nanoengineered chiral cavity (currently produced using techniques akin to DVD manufacturing), it eliminates the need for complex chemical steps involving stereostructural control, potentially simplifying the synthesis of chiral materials.

## Methods and Experimental Section

### Chiral Nanocavity Fabrication

The 1000/1500 nm nanocavity templated substrates were realized using injection molding (ENGEL), using a previously established method.^50^ The master shim for this was made by using e-beam lithography. In a nutshell, 100 nm of PMMA was spin-coated onto a Si wafer, baked for an hour at 180 °C, and patterned using a VB6 UHR EWF lithography tool (Vistec). The exposed resist was developed in IPA and methyl isobutyl ketone, MIBK (3:1 ratio) for 60 s followed by electroplating Ni onto the surface. The patterned Ni was removed from the wafer to provide the shim that was then used as the master in a tool placed in the injection molder.

The polycarbonate pellets are thermally heated and pushed into the tool to create small plastic slides with nanostructures indented on the surface. These slides are then coated with 100 nm of Au in an e-beam evaporator at a rate of ≈0.3 nm s^–1^. When gold is evaporated onto the surface, it takes the shape of the indentation and forms a hybrid plasmonic structure, constituting an inverse structure at the top and a solid one at the bottom.

### Thin Film Preparation

200 mg portion of PMMA was weighed in a vial in a regular atmosphere before being inserted into the glovebox (MBraun). Once in the glovebox, 4 mL of toluene was pipetted into the vial of PMMA and then left to dissolve on a hot plate for an hour at 50 °C. In the meantime, a high-concentration stock solution of MHeB14 was made up in the glovebox using toluene by pipetting 2 mL of toluene into a vial followed by some scrapings of the yellow MHeB14 crystals. The resultant mixture was shaken vigorously and left for around half an hour to ensure it was well and truly dissolved in toluene to give a vivid yellow solution.

Once the PMMA was fully dissolved, the MHeB14 and PMMA solutions were then used to make up a 50% (w/w) MHeB14 solution for spinning. The substrate was placed onto the chuck and spun for 60 s at a rate of 2000 rpm. In these 60 s, a pipet was used to take up 100 μL of the respective solution from the vial and thus deposited onto the substrate while spinning.

### Refractive Index Measurement

M-2000XI (JA Wollam) was used for measuring the RI of the films. The amplitude psi (Ψ) and phase delta (Δ) were collected at an incident angle from 45 to 75° at a difference of 5°. The obtained data was fitted using CompleteEase software. The dye-doped PMMA was modeled as B-spline with a general oscillator as the starting material. The real and imaginary parts of refractive index for various dye concentrations are shown in Figure S5.

### Optical Measurement

The PL, reflectance, and Fourier plane measurements were carried out using a home-built 30 mm cage-rod setup. For PL and Fourier experiment, CW diode laser (λ_max_= 404 nm; L404P400M; Thor laboratories) operating at a constant current of 180 mA was collimated using a lens and redirected into the pair of polarizers with the help of a left turning mirror, as shown in Figure S6(a). The two polarizers were used to control the power and polarization of the incident beam. The linearly polarized beam was then directed toward the sample using a 50:50 beam splitter and focused onto the sample by a 10× objective (NA = 0.3) to a spot size of 350 μm. The input power at the sample was ∼70 μW. The reflected light was collected using the same objective and passed through a combination of quarter wave plate and analyzer. The circularly polarized emission was detected with ±45° of the analyzer position while the Quarter Wave plate was kept at 0°. The 90:10 beam splitter was used to send 10 and 90% of the emitted light to the Camera and Spectrograph (SR-303i-B, Andor Technology), respectively. The lens was used to focus the light to the optical fiber connected to the spectrometer, and the long pass filter (cutoff wavelength ∼450 nm) was used to stop the laser beam from reaching and saturating the detector. The EMCCD camera (DU970P-BVF, Andor Technology) was used to acquire the signal with the acquisition time of 0.1, and 500 spectrum was added for each measurement. External light source was used to locate the 1000/1500 nm metafilm with the help of the bright field camera.

For the broadband experiment, the same setup was used except replacing the diode laser with the broadband light (HL-2000-FHA; Ocean Optics) source and removing the left turning prism, as shown in Figure S6(b).

For Fourier plane analysis, the spectrometer setup was slightly modified as shown in Figure S6(c). Black and white CMOS camera (Ximea) with an exposure time of 0.25 s was placed at the focal plane of the third lens. The band-pass filter centered around 560 nm with an fwhm of 10 nm was used and 30 images were accumulated to get the final image.

### FLIM Measurement

Microscopy experiments were conducted using a Zeiss LSM710 laser scanning confocal microscope equipped with a 10× objective (NA = 0.25), offering a spatial resolution of 0.8 μm. Areas of approximately 392 × 386 μm^2^ were mapped with a total acquisition time of 20 s per frame. For FLIM measurements, the system employed a 405 nm laser pulsed at 20 MHz and a Becker–Hickl Simple–Tau detection system for time-correlated single photon counting (TCSPC). FLIM images comprised 256 × 256 pixels acquired at an average photon flux of ∼1 × 10^6^ photons s^–1^. Data were accumulated to achieve sufficient photon counts for fitting purposes (approximately 1500 photons per pixel followed by 9-pixel binning applied as a sliding average).

Data analysis was performed using SPCImage software (Becker & Hickl). To obtain fluorescence lifetime distributions, the accumulated fluorescence decay curves in each pixel of the scanned area were fitted with an appropriate model. In all measurements, a double-exponential decay model was used, yielding a lifetime for each pixel in the image. Fluorescence decay curves were collected to a total delay time of ∼50 ns, ensuring a good fit (χ^2^ ≈ 1) and accurate background noise-level determination to prevent lifetime underestimation.

### Simulation

We have conducted a series of numerical simulations of Maxwell’s equations by using a finite-element approach with COMSOL V6Multiphysics software with the Wave optics module. The Shuriken’s dimensions were obtained from the scanning electron microscopy (SEM) image, and 200-nm-thick PMMA was used on the top. The excitation port was placed 850 nm above the top of the gold surface. Floquet periodic boundary conditions were applied on the side walls, and a 200 nm perfectly matched layer was used outside excitation, and receiving ports absorb all of the wave coming out of the simulated domain. The refractive index values used for Au, PMMA, and PMMA doped with the dye were extracted from ellipsometry, and values of Zhang et al., given in the COMSOL library, were used for polycarbonate. Meshing was done using a Physics-controlled mesh. The model used in the simulation is shown in Figure S7.

Angle-resolved Fourier plane simulations were conducted by utilizing the reciprocity theorem. Left- and right-handed circularly polarized light was directed onto the doped substrate at radial angles ranging from 0 to 64° at an interval of 4° and azimuthal angles spanning from 0 to 180° with an interval of 5°. For each incident angle, the volume-integrated magnitude of the electric field was computed.

For single dipole simulation, a point source (electric dipole) was displaced in the middle of the cavity. To mimic the experimental conditions, the emission intensity of a dipole coupled to the cavity for three polarizations (*X*, *Y*, and *Z*) was computed and then averaged. The electric current dipole moment equal to 1 A·m was used and to decompose the emission intensity in two distinct polarizations, right- and left-handed, the following formalism was undertaken
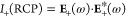

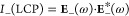
where **E**_+_(ω) and **E**_–_(ω) electric fields stand for right- and left-handed polarizations. The polarization-defined electric field of the emitted light is expressed as
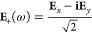

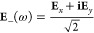


In the 2D eigenmode analysis, periodic boundary conditions were applied to all four sides of the 2D domain, which contained a shuriken-shaped dye encircled by gold. The linearization point was set at 570 nm.

## Supporting Information Available

Synthesis and material characterization (NMR spectra) of the MHeB14 dye; figures showing the refractive index of the films calculated from ellipsometry; characterization setup; COMSOL model; electric-field intensity (left) and optical chirality (right) for MHeB14-doped 1000 nm metafilms; circularly polarized relative reflectance of metafilms; CPL(gCPL) and CD (gCD) spectra; experimental resolved LCP and RCP emission along with dichroism (LCP–RCP) of MHeB14-doped RH 1000 and 1500 nm metafilms; simulated angle-resolved LCP and RCP emissions along with dichroism (LCP–RCP) of MHeB14-doped LH 1000 nm metafilm at an excitation energy of 3.06 eV; spatial PL map of LH 1000 MHeB14-doped metafilm; eigenmodes for 1000/1500 nm doped metafilm; lattice mode orders and respective position for 1000 and 1500 nm periodic structure; and Gaussian fitting parameters of three luminescence bands transitions of MHeB14 on a flat gold substrate (PDF)
