# Supplementary material for: Electromagnetic Enantiomer: Chiral Nanophotonic Cavities for Inducing Chemical Asymmetry
Source: ACS Nano. 2024 Aug 6;18(33):22220–32. doi: 10.1021/acsnano.4c05861 (PMC11342365; doi:10.1021/acsnano.4c05861)
Supplement: Supplementary file 1 — nn4c05861_si_001.pdf [file nn4c05861_si_001.pdf]

The Electromagnetic Enantiomer: Chiral Nanophotonic Cavities for Inducing Chemical Asymmetry.

Rahul Kumar[1], Ben Trodden[1], Anastasia Klimash[1], Manon Bousquet[1], Shailendra K Chaubey[1], Nicola J. Fairbairn[1], Ben A. Russell[1], Klaas Wynne[1], Affar S. Karimullah[1], Nikolaj Gadegaard[2], Peter J. Skabara[1], Gordon J. Hedley[1], Artur Movsesyan[3,4], Shun Hashiyada[5,6], Alexander O. Govorov [3] and Malcolm Kadodwala[1]**

[1] School of Chemistry, Joseph Black Building, University of Glasgow, Glasgow, G12 8QQ, UK

[2] James Watt School of Engineering, Rankine Building, University of Glasgow, Glasgow G12 8LT, U.K

[3]Innovative Photon Manipulation Research Team, RIKEN Center for Advanced Photonics, 2-1 Hirosawa, Wako, Saitama 351-0198, Japan

[4]Department of Electrical, Electronic, and Communication Engineering, Chuo University, 1-13-27 Kasuga, Bunkyo-Ku, Tokyo 112-8551, Japan.

[5]Department of Physics and Astronomy and Nano scale and Quantum Phenomena Institute, Ohio University, Athens, OH 45701, USA

[6]Institute of Fundamental and Frontier Sciences, University of Electronic Science and Technology of China, Chengdu 610056, China.

MHeB14 synthesis & Characterization:

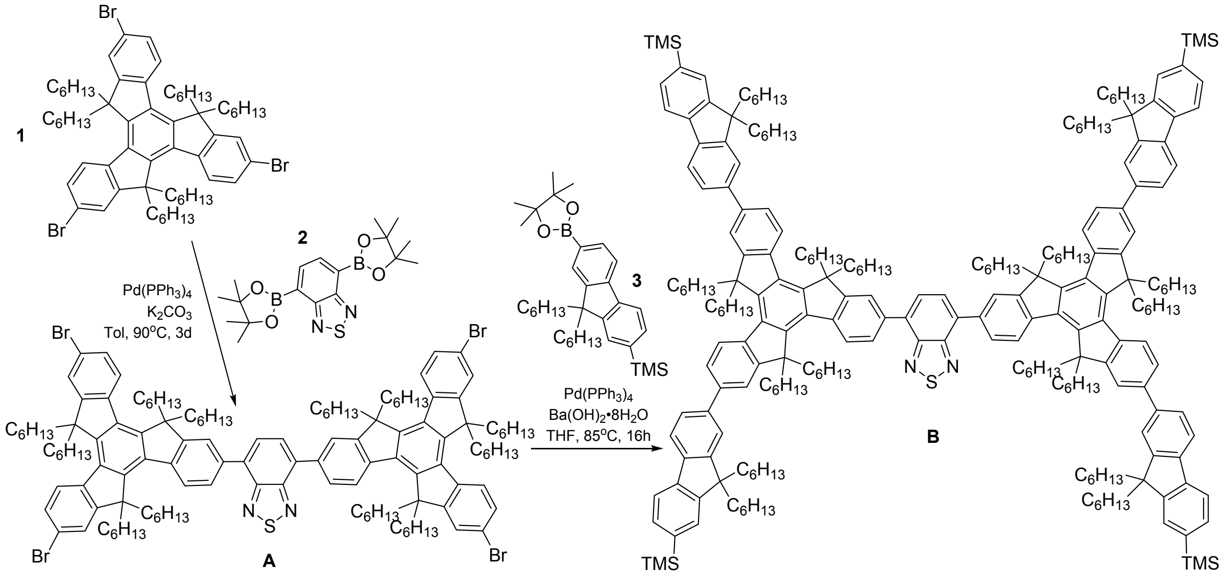

Scheme S1. *Synthesis of the MHeB14 (represented as B)*

Compound A was synthesised via Suzuki-Miyaura coupling between bromide 1 and 2,1,3-benzothiadiazole-4,7-bis(boronic acid pinacol ester) 2 (Scheme S1). Truxene derivative 1 was used in large excess (7:1 ratio) in order to minimise side polymerisation reactions and achieve higher selectivity. However, the best yield for the reaction is still as low as 18 % due to the selectivity issues. The target compound B was similarly synthesised via Suzuki-Miyaura coupling of A with 9,9-dihexyl-2-trimethylsilane-7-boronic ester-9H-fluorene 3, giving the product in 63% yield.

Experimental details:

Materials: Unless stated otherwise, all reagents were purchased from Sigma Aldrich, TCI, Alfa Aesar or Fluorochem and used without further purification. Compound **1** was prepared according to the previously reported procedure. [1]

Instrumentation and Analytical Techniques: Melting points were determined using a Stuart Scientific SMP1 Melting Point Apparatus or Stuart SMP50 Automatic Melting Point Apparatus. ^{1}H and ^{13}C NMR spectra were recorded on a Bruker AVIII 400 apparatus at 400 MHz and 100 MHz, respectively, or a Bruker AV500 at 500 MHz and 125 MHz, respectively. NMR data are presented in the following order: chemical shifts (δ) in ppm; multiplicity as singlet (s), doublet (d), triplet (t), quartet (q), multiplet (m); coupling constants (*J*) in Hz. Multiplets are reported over the range they appeared (in ppm). MS MALDI-TOF analyses were run on a Shimadzu Axima-CFR spectrometer (mass range 20-150000Da); Thermogravimetric analyses (TGA) were performed on a Perkin-Elmer Thermogravimetric Analyser TGA7 or on a NETZSCH TG 209 F3 - Tarsus thermogravimeter under a

constant flow of Argon or Nitrogen (20 mL/min). The temperature was increased at a rate of 10 °C/min from 40 °C to 500 °C. Differential scanning calorimetry (DSC) was carried out on a TA Instruments Q1000 with a RC-90 refrigerated cooling unit attached or on a NETZSCH DSC 214 – Polyma differential scanning calorimeter.

Synthesis:

Compound A: 4,7-Bis(7,12-dibromo-5,5,10,10,15,15-hexahexyl-10,15-dihydro-5H-diindeno[1,2-a:1',2'-c]fluoren-2-yl)benzo[c][1,2,5]thiadiazole

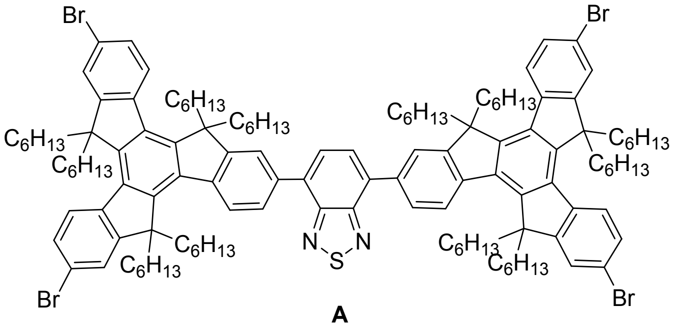

A

Scheme S2: *Molecular Structure of compound A*

A mixture of tribromohexahexyltruxene **1** (13 g, 12 mmol, 7 eq.), tetrakis(triphenylphosphine)palladium (0) (0.8 g, 0.68 mmol, 0.4 eq.) and 2,1,3-benzothiadiazole-4,7-bis(boronic acid pinacol ester) **2** (0.665 g, 1.7 mmol, 1 eq.) was dried under reduced pressure for 30 min, then dissolved in dry degassed toluene. An aqueous degassed solution of K_2CO_3 (2M, 4.28 mL) was added and the mixture was stirred at 100°C for 3 days. The mixture was washed with water (200 mL), the toluene layer was separated, and aqueous phase was extracted with dichloromethane (2×200 mL). The organic phases were combined, dried over $MgSO_4$ and concentrated under reduced pressure. The crude mixture was purified by column chromatography on silica gel. The starting material was first eluted with hexane, then the product was eluted with a hexane : toluene (5:1) mixture. Pure compound **A** was obtained as a yellow-green powder (660 mg, 0.3 mmol, 18%): mp 124-126 °C; MS (MALDI-TOF) calculated for $C_{132}H_{176}Br_4N_2S$ 2137.03, found 2141.97 [M^+]; ^1H NMR (400 MHz, $CDCl_3$) δ 8.53 (d, J = 8.9 Hz, 2H), 8.29 (d, J = 8.6 Hz, 2H), 8.23 (d, J = 8.6 Hz, 2H), 8.20 – 8.18 (m, 4H), 8.05 (s, 2H), 7.63 (dd, J = 7.7, 1.9 Hz, 4H), 7.57 (ddd, J = 8.3, 5.0, 1.9 Hz, 4H), 3.10 – 3.03 (m, 4H), 2.97 – 2.87 (m, 8H), 2.33 – 2.21 (m, 4H), 2.14 – 2.04 (m, 8H), 1.13 – 0.79 (m, 72H), 0.78 – 0.61 (m, 36H), 0.55 (dt, J = 20.3, 6.3 Hz, 24H). ^{13}C NMR ($CDCl_3$) δ 155.54, 155.43, 153.90, 153.48, 145.39, 144.70, 144.25, 139.60, 138.64, 138.56, 137.75, 137.21, 137.04, 135.18, 132.64, 128.83, 127.54, 126.90, 125.48, 125.39, 125.06, 125.01, 124.24, 122.62, 120.43, 55.63, 55.50, 55.37, 36.49, 36.40, 31.04, 30.97, 29.01, 28.96, 28.91, 23.50, 23.42, 21.80, 21.77, 13.40. Elemental Analysis: Found: C, 73.80, H, 8.34, N, 1.30, Expected: C, 74.00, H, 8.28, N, 1.31.

Compound B: 4,7-bis(7,12-bis(9,9-dihexyl-7-(trimethylsilyl)-9H-fluoren-2-yl)-5,5,10,10,15,15-hexahexyl-10,15-dihydro-5H-diindeno[1,2-a:1',2'-c]fluoren-2-yl)benzo[c][1,2,5]thiadiazole

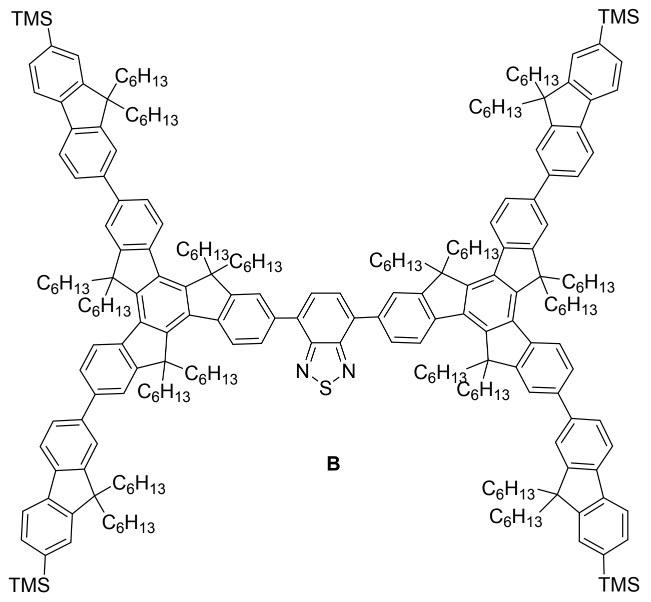

Scheme S3: *Molecular Structure of compound B*

The mixture of **A** (202mg, 0.098mmol, 1eq), and (9,9-dihexyl-7-(4,4,5,5-tetramethyl-1,3,2-dioxaborolan-2-yl)-9H-fluoren-2-yl)trimethylsilane **3** (781mg, 1.462mmol, 15eq), with tetrakis(triphenylphosphine)palladium(0) (56mg, 0.049mmol, 0.5eq) and $Ba(OH)_2 \cdot 8H_2O$ (769mg, 2.437mmol, 25eq) was dried under vacuum during 30 min and then dissolved in dry degassed THF (15mL). Degassed water was added (1.61mL) and the mixture was heated at 85 °C overnight. The crude mixture was washed with water and the aqueous layer was extracted with toluene, organic layers were combined, dried with $MgSO_4$ and evaporated under vacuum. The product was purified by column chromatography (silica gel, hexane, then 10:1 hexane/toluene) to afford a bright yellow powder (231 mg, 68 %) mp 114-128 °C; MS (MALDI-TOF) calculated for $C_{244}H_{340}N_2SSi_4$ 3442.55, found 3363.79 [M+(– C_6H_{13})]; ^{1}H NMR (400 MHz, CDCl3) δ 8.60 (d, J = 8.0 Hz, 2H), 8.51 (d, J = 8.1 Hz, 4H), 8.25 – 8.16 (m, 4H), 8.06 (s, 2H), 7.84 (d, J = 7.8 Hz, 4H), 7.80 (s, 6H), 7.77 – 7.74 (m, 14H), 7.57 – 7.48 (m, 8H), 3.15 – 3.04 (m, 12H), 2.38 – 2.16 (m, 12H), 2.07 (t, J = 8.1 Hz, 16H), 1.22 – 1.07 (m, 48H), 1.07 – 0.85 (m, 72H), 0.85 – 0.54 (m, 100H), 0.36 – 0.31 (m, 39H). ^{13}C NMR (CDCl3) δ 151.54, 151.29, 148.88, 147.31, 142.62, 142.54, 139.88, 138.61, 137.80, 137.53, 137.37, 136.68, 136.13, 135.34, 135.29, 135.19, 128.99, 125.19, 124.77, 124.44, 123.36, 123.09, 122.39, 122.07, 118.45, 117.66, 117.61, 117.20, 116.14, 53.03, 52.28, 50.53, 37.36, 34.29, 28.71, 28.51, 26.83, 26.74, 21.25, 20.87, 19.78, 19.64, 19.51, 19.47, 11.24, 11.13, 11.07, 11.05, -1.86, -3.72.

NMR characterization of compound A and B are shown in figures S1-S4.

NMR spectra

Compound A:

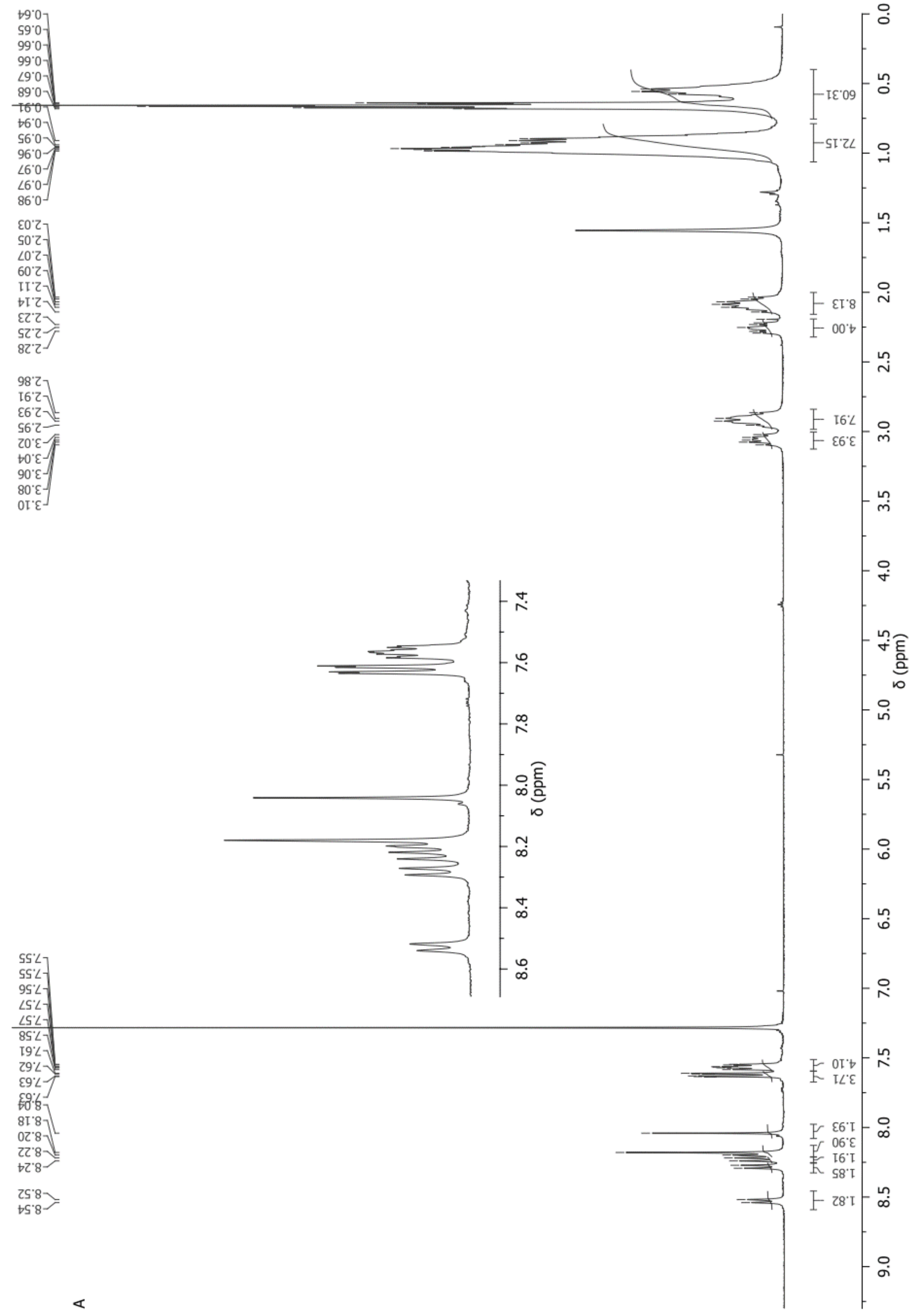

Figure S1 *^{1}H NMR spectrum of compound A in $CDCl_3$, 400MHz, 293 K.*

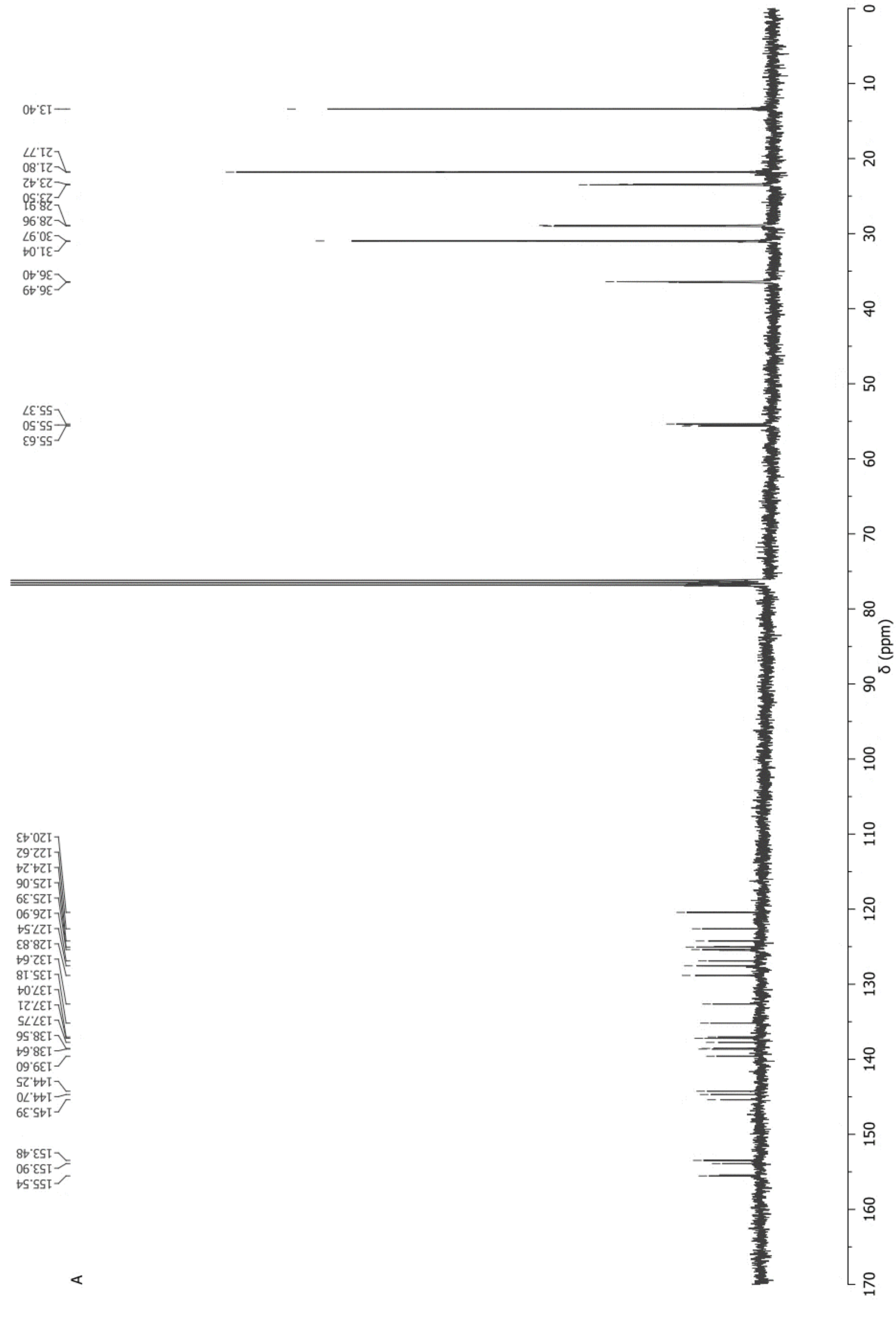

Figure S2 ^{13}*C NMR of compound A in CDCl$_3$, 400MHz, 293 K.*

Compound B:

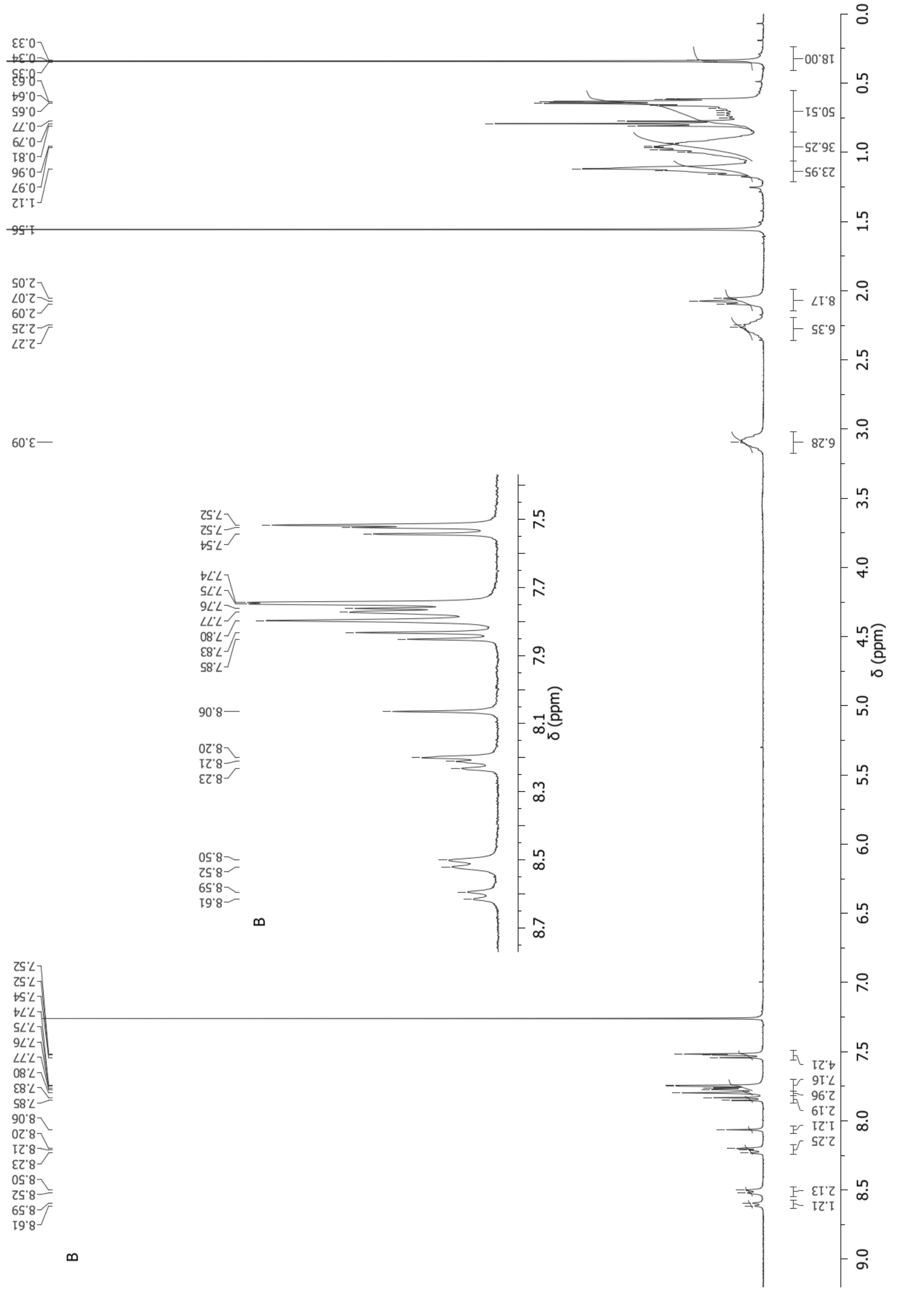

Figure S3 *^{1}H NMR spectrum of compound B in $CDCl_3$, 400MHz, 293 K.*

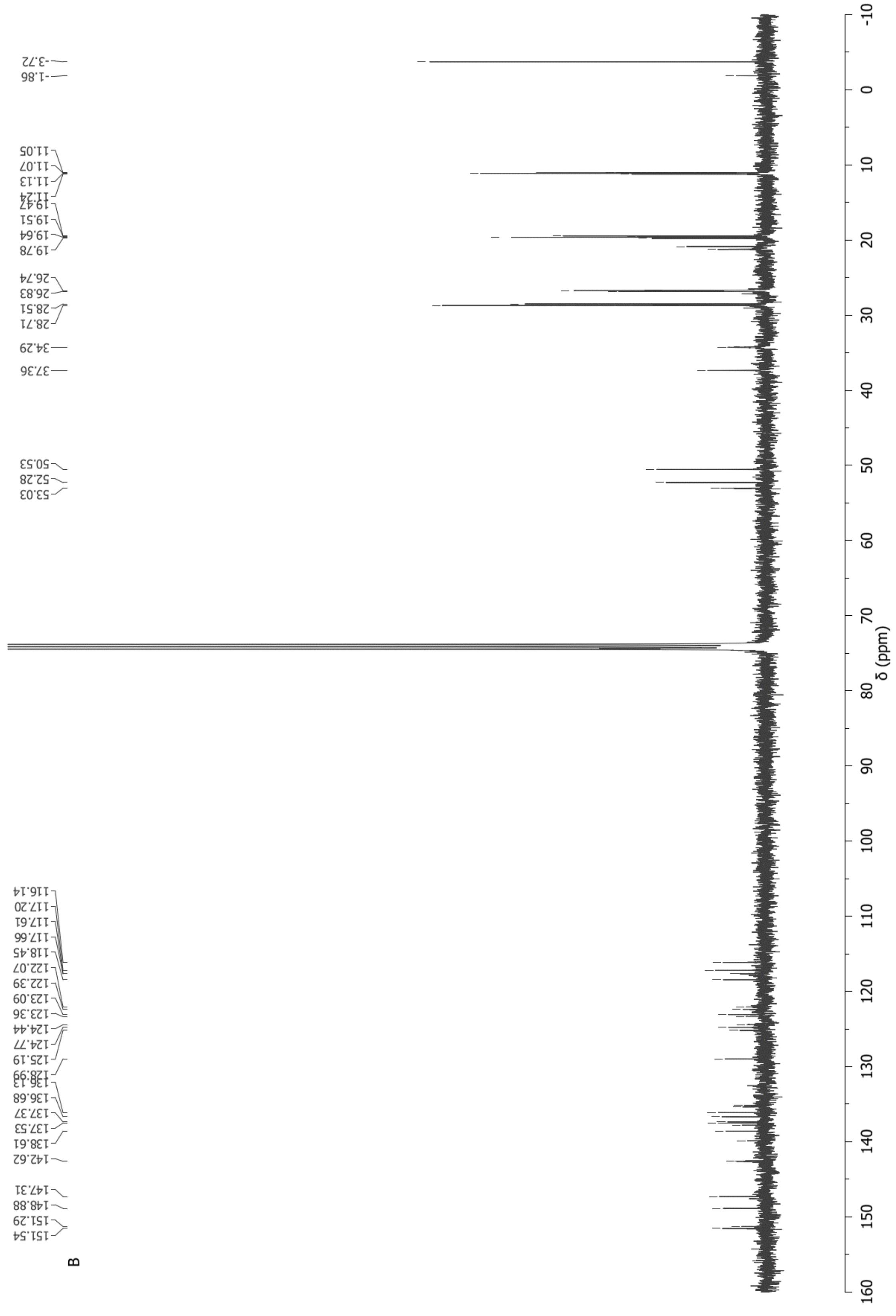

Figure S4 ^{13}*C NMR of compound B in CDCl*$_3$*, 400MHz, 293 K.*

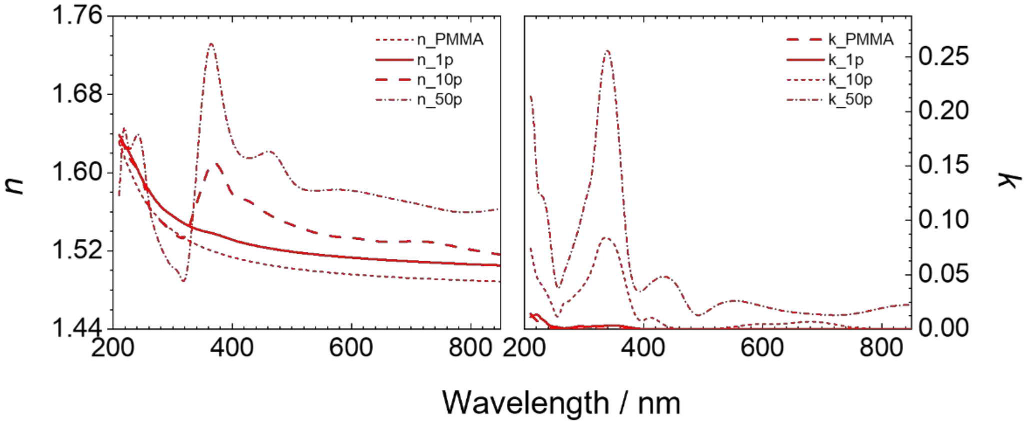

Figure S5: *RI of the films calculated from ellipsometry (a) Real part (b) Imaginary part*

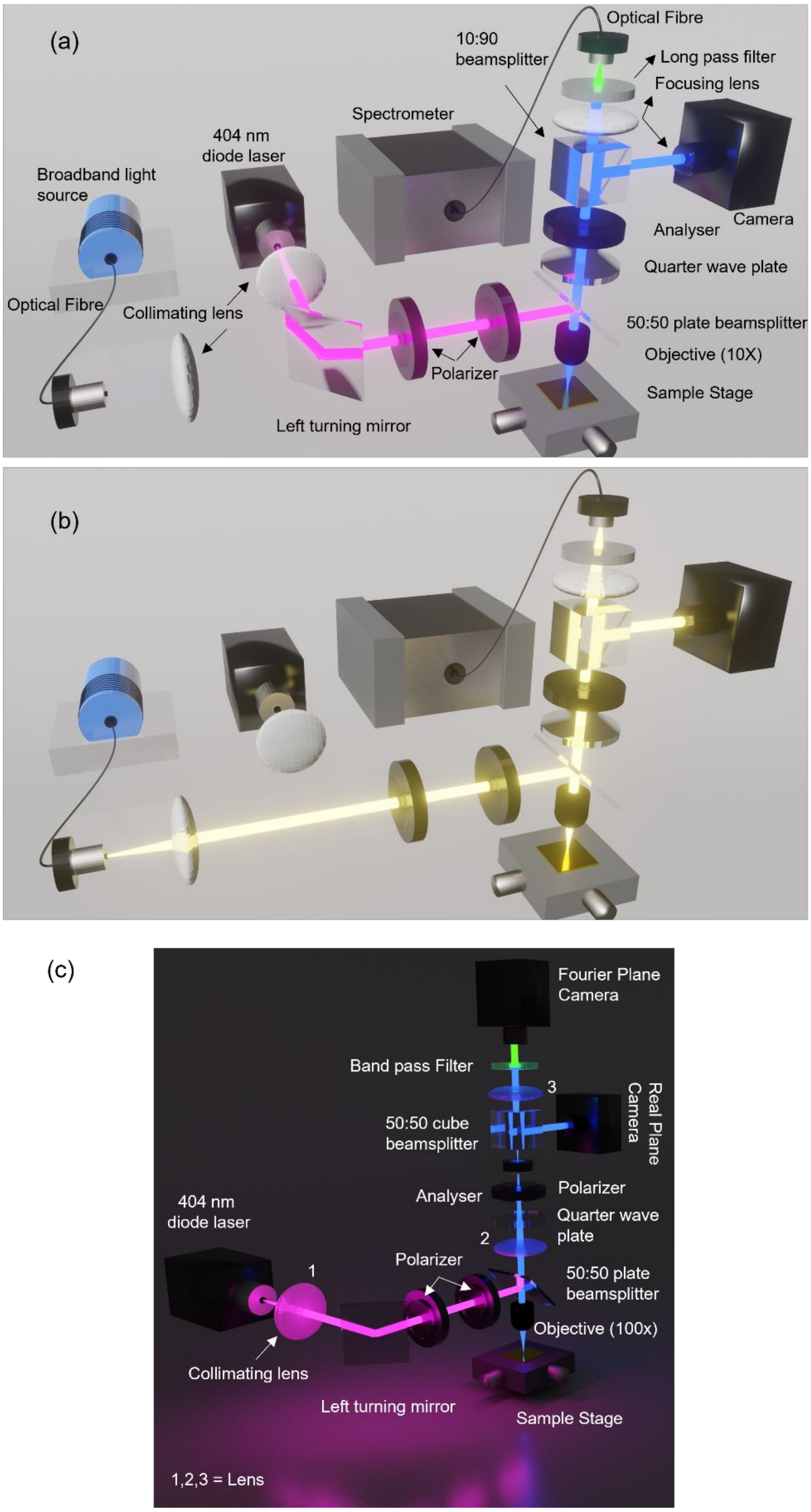

Figure S6 *Schematic showing characterization setup for (a) PL (b) Broadband (c) Fourier plane measurement*

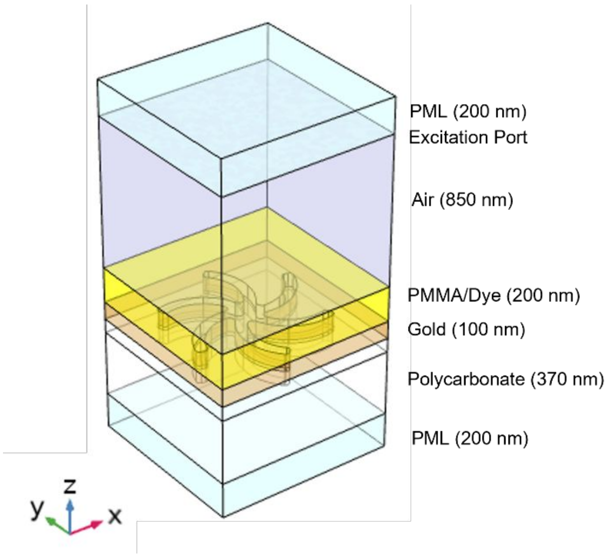

Figure S7 *COMSOL model used for single dipole and angle resolved Fourier plane simulation.*

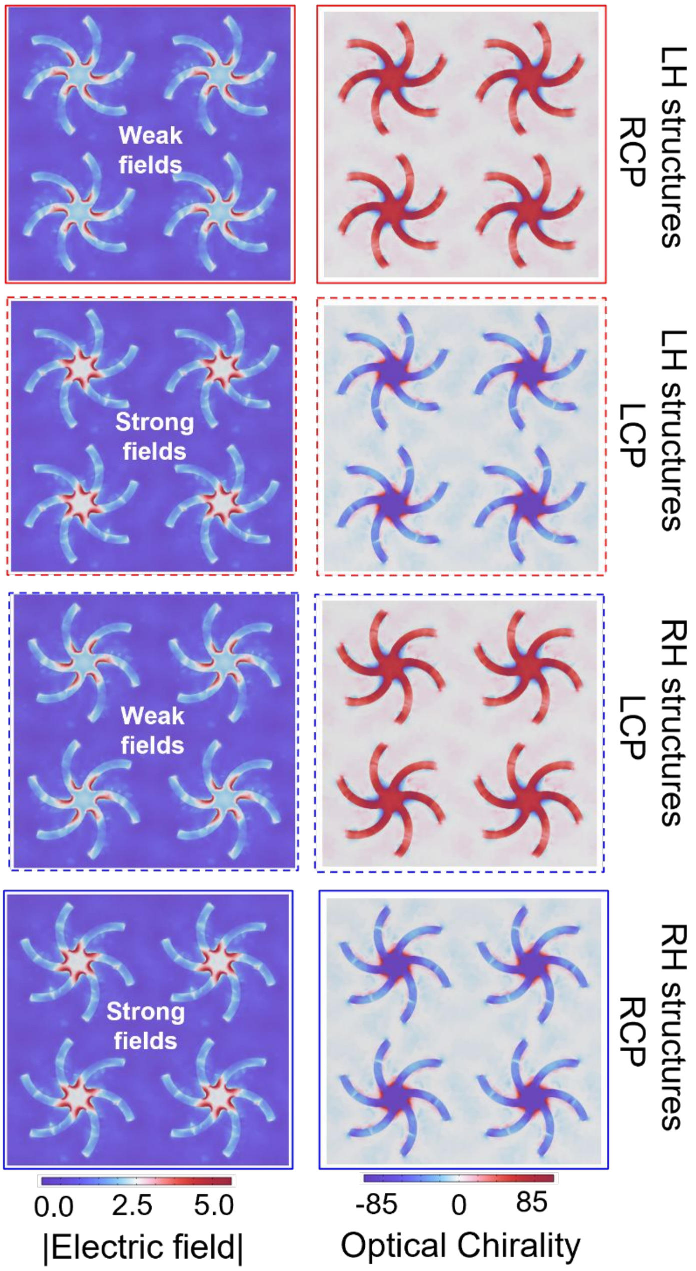

Figure S8 *The simulated relative electric-field intensity (left) and Optical Chirality (right) for MHeB14 doped 1000 nm metafilm. The field plots are at the centre of the cavity for eigen mode A (2.167eV) and have been normalised with corresponding values of the flat gold. The strong field confinement is observed for matched combination of metafilm handedness and input light helicity i.e., LH structures/LCP and RH structures/RCP. The incident E-field used in simulation are left and right circularly polarized.*

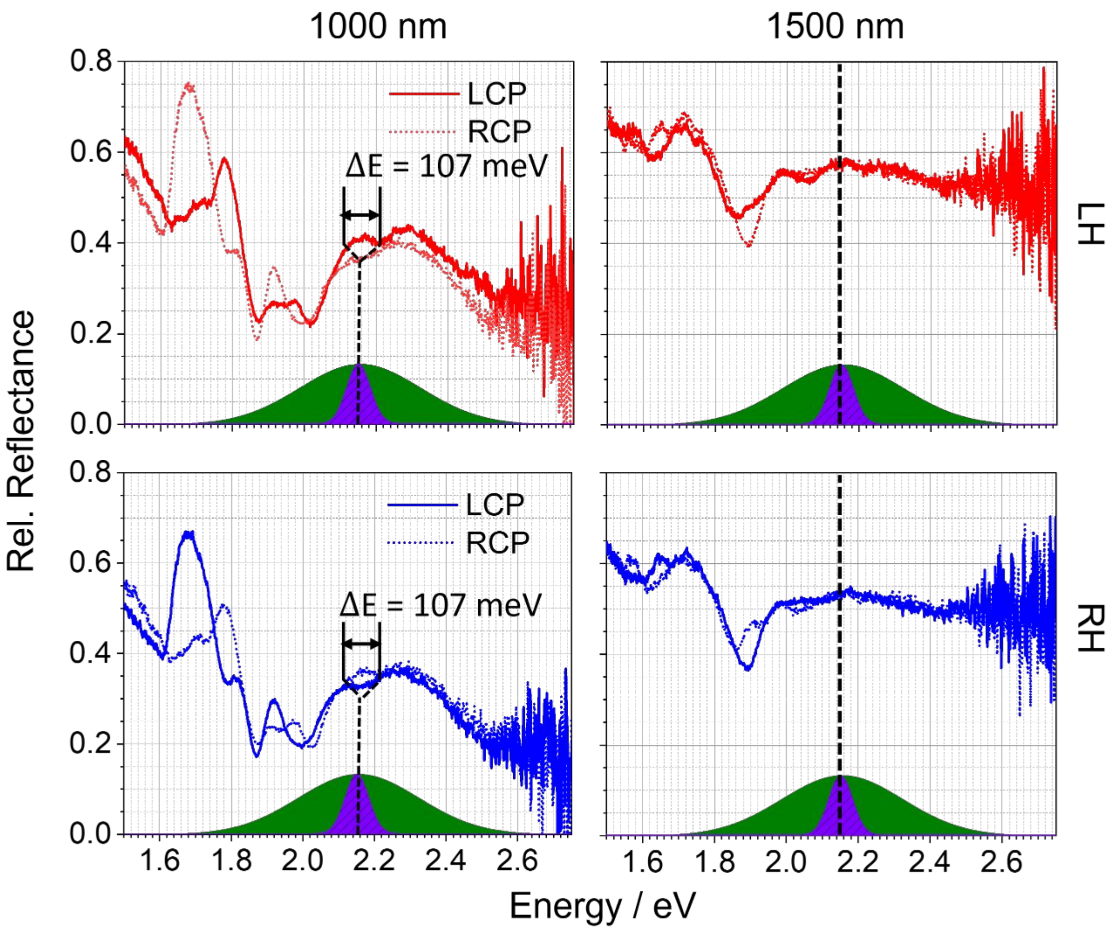

Figure S9 *The circularly polarised relative reflectance LH (red; top) and RH (blue; bottom) for 1000 nm (left) 1500 nm (right) metafilm. The dashed line and solid line respectively show LCP and RCP reflectance from the structures. The olive and blue coloured solid dash filled spectra correspond to luminescence Band I of molecules on flat gold and dark eigenmode A of nanocavity. The dashed black vertical lines are guidance for eyes, highlighting the correspondence between reflectance peaks, maxima of dark eigenmode and luminescence band I.* ΔE *shows the rabi splitting of peak for matched combination.*

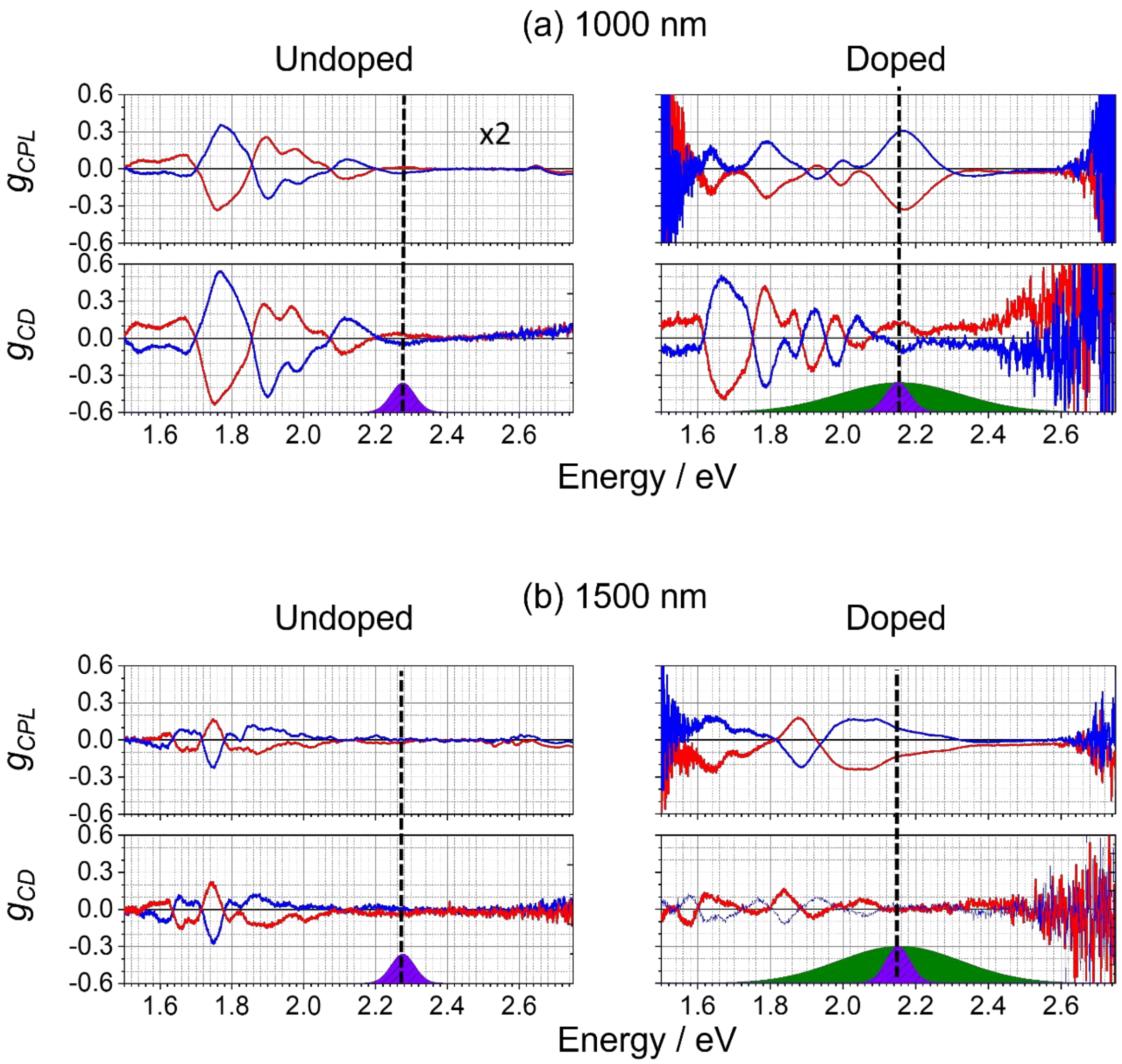

Figure S10 *CPL(gCPL) and CD (gCD) spectra for 1000 and 1500 nm metafilm. The chiroptical math is done with* $g_{CD,refl} = 2\frac{I_{LCP,refl}-I_{RCP,refl}}{I_{LCP,refl}+I_{RCP,refl}}$, *where* $I_{LCP,\ refl}$ *and* $I_{RCP,\ refl}$ *are the intensities of reflected LCP and RCP light.* $g_{CPL,emit} = 2\frac{I_{LCP,emit}-I_{RCP,emit}}{I_{LCP,emit}+I_{RCP,emit}}$ *where* $I_{LCP,\ emit}$ *and* $I_{RCP,\ emit}$ *are the intensities of emitted LCP and RCP light. The olive and blue coloured solid dash filled spectra correspond to luminescence Band I of molecules on flat gold and dark eigenmode A of nanocavity. The dashed black vertical lines are guidance for eyes, highlighting the correspondence between CD/CPL, maxima of dark eigenmode A and/or luminescence band I.*

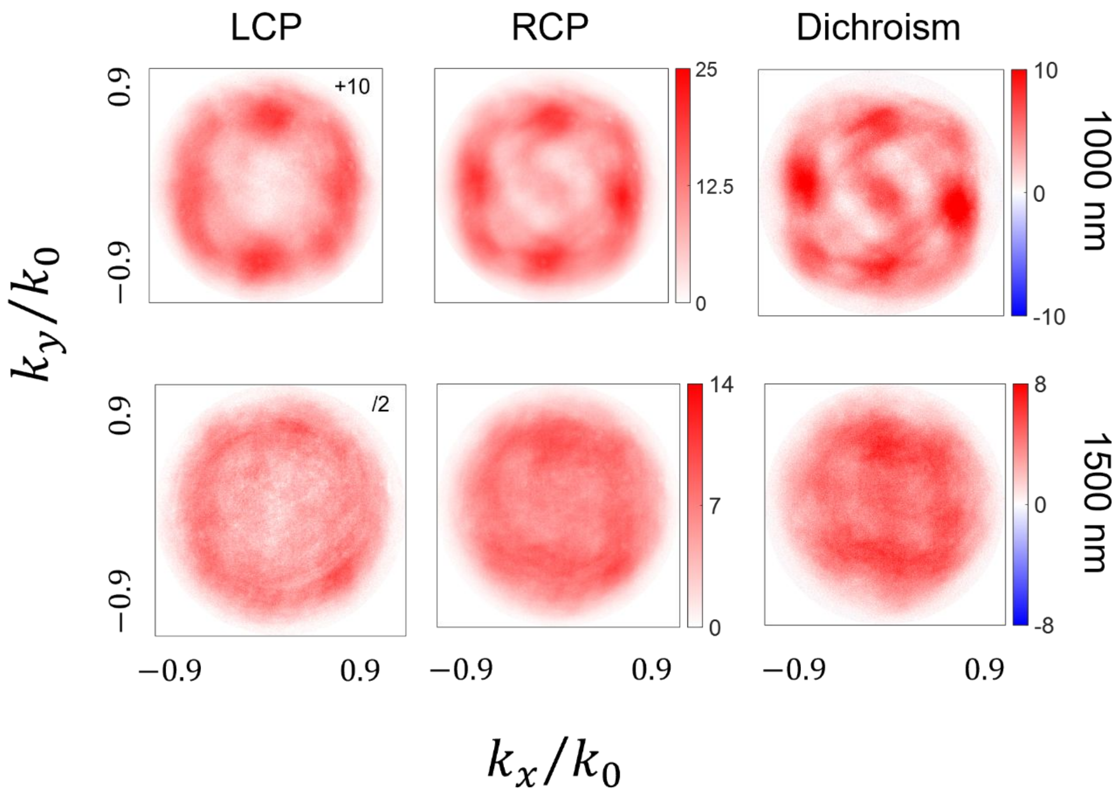

Figure S11 *Experimental resolved LCP and RCP emission along with Dichroism (LCP-RCP) of MHeB14 doped RH 1000 & 1500nm metafilm.*

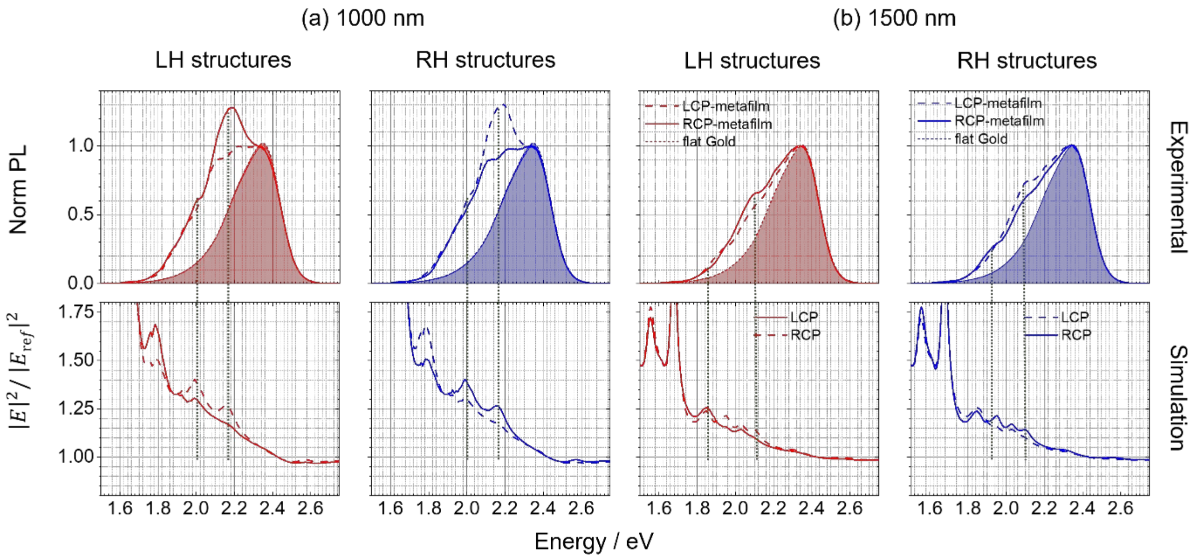

Figure S12 (a), (b) *Normalised -PL (top) and the simulated relative electric-field intensity with respect to flat gold (bottom) in 200 nm thick volume for 1000 nm and 1500 nm, MHeB14 doped metafilms. The black vertical lines are guidance for eyes, highlighting the correspondence between PL and electric-field intensity peaks. The (red and blue) dashed line and solid line respectively show the LCP and RCP PL emission from the structures. The spectra which are solid filled in red and blue show LCP and RCP PL emission of MHeB14 when present on the flat gold. All PL spectra have been self-normalised to value at* $2.33\ eV$. *In simulation, the dashed and solid line respectively shows relative electric-field intensity when the incident E-field is left and right circularly polarized.*

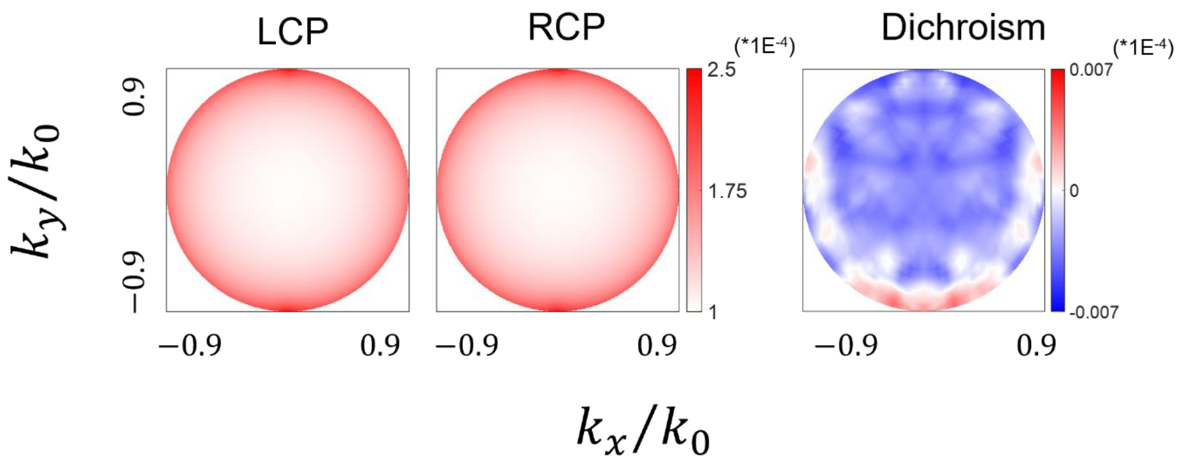

Figure S13 *Simulated angle-resolved LCP and RCP emissions, along with dichroism (LCP-RCP), of MHeB14-doped LH 1000 nm metafilm at an excitation energy of 3.06 eV. The colours represent the volume-integrated E-field magnitude in the 200 nm film. Compared to the emission pattern in Figure 6 of the main article, the LCP and RCP emissions are similar and uniform, lacking any distinct pattern. Consequently, the dichroism is two orders of magnitude lower than what was observed at the emission energy and does not follow C2 symmetry, indicating noise related to the simulation.*

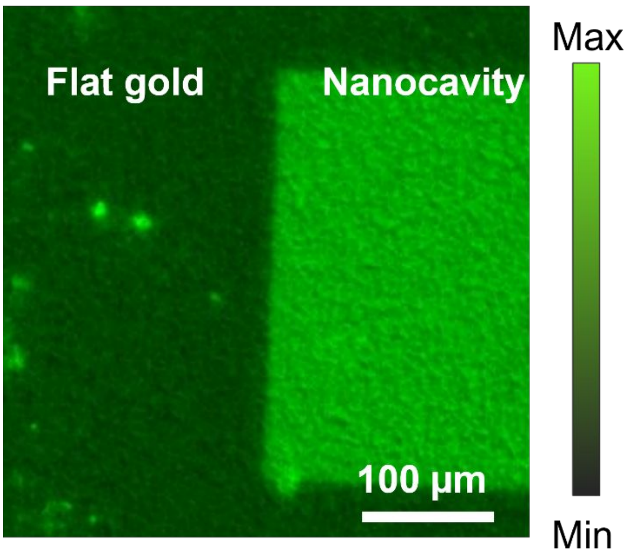

Figure S14 *Spatial PL map of LH 1000 MHeB14-doped metafilm. The PL intensity of dye on the top of nanocavity is higher than that on flat gold.*

Table S1 *Gaussian fitting parameters of three luminescence bands transitions of MHeB14 on a flat gold substrate.*

	**Luminescence Band I**	**Luminescence Band II**	**Luminescence Band III**
**Equation**	y=y0 + (A/(w*sqrt(pi/2)))*exp(-2*((x-xc)/w)^2)		
**y0**	13.2 ± 8.086E-11	13.2 ± 1.979E-10	13.2 ± 9.9187E-11
**xc**	2.155 ± 4.399E-16	2.286 ± 2.869E-16	2.385 ± 1.685E-16
**w**	0.345 ± 9.507E-16	0.219 ± 6.0004E-16	0.128 ± 3.452E-16
**A**	45315.009 ± 1.253E-10	96864.416 ± 2.477E-10	39343.570 ± 9.516E-11

Table S2 *Eigenmodes for 1000 nm/1500 nm doped metafilm*

**Eigen Mode (eV)**	**Width (eV)**	**Nature**	**E_y (V/m)**
1.977	0.040	Non-dipolar character	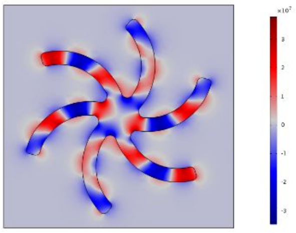
2.078	0.066	Dipolar character	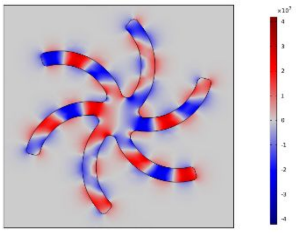
2.156	0.090	Non-dipolar character	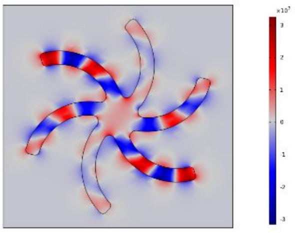
2.191	0.106	Non-dipolar character	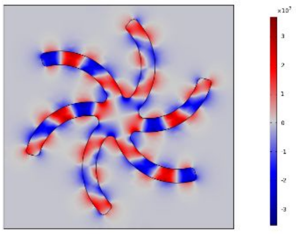
2.245	0.126	Dipolar character	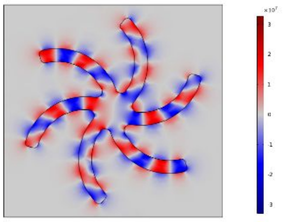

Supplementary Table 2 Lattice mode Orders and respective position for 1000 nm and 1500 nm periodic structure.

Lattice Mode Orders (n_x, n_y)	Position in eV (1000 nm periodicity)	Position in eV (1500 nm periodicity)
0,1	0.827	0.551
1,1	1.170	0.779
2,0	1.653	1.102
2,1	1.850	1.232
2,2	2.339	1.559
2,3	2.980	1.987
3,3	3.512	2.339

Lattice contact for homogenous medium is calculated using following equation

$$\lambda_{\mathrm{LP}(i,j)} = P \cdot n \frac{\sqrt{(i^2+j^2) - j^2 \sin^2\theta} \pm i \cdot \sin\theta}{i^2+j^2}$$

where P is the lattice constant, i and j are integers, θ is the angle of incidence of the impinging light, and n is the refractive index of the optical medium.

Table S3 Gaussian fitting parameters of three luminescence bands transitions of MHeB14 on a flat gold substrate.

	Luminescence Band I	Luminescence Band II	Luminescence Band III
**Equation**	y=y0 + (A/(w*sqrt(pi/2)))*exp(-2*((x-xc)/w)^2)		
**y0**	13.2 ± 8.086E-11	13.2 ± 1.979E-10	13.2 ± 9.9187E-11
**xc**	2.155 ± 4.399E-16	2.286 ± 2.869E-16	2.385 ± 1.685E-16
**w**	0.345 ± 9.507E-16	0.219 ± 6.0004E-16	0.128 ± 3.452E-16
**A**	45315.009 ± 1.253E-10	96864.416 ± 2.477E-10	39343.570 ± 9.516E-11

Reference

[1] Kanibolotsky, A. L.; Berridge, R.; Skabara, P. J.; Perepichka, I. F.; Bradley, D. D. C.; Koeberg, M. Synthesis and Properties of Monodisperse Oligofluorene-Functionalized Truxenes: Highly Fluorescent Star-Shaped Architectures. *J. Am. Chem. Soc.* **2004**, *126* (42), 13695–13702. https://doi.org/10.1021/ja039228n.
